# Partial Deletion of *Cxcl12* from Hippocampal Cajal–Retzius Cells Does Not Disrupt Dentate Gyrus Development or Neurobehaviors

**DOI:** 10.1523/ENEURO.0245-25.2025

**Published:** 2026-01-07

**Authors:** Rebekah van Bruggen, Karla Manzanet Freyre, Sangeetha Vasanthkumar, Mi Wang, Qiumin Tan

**Affiliations:** ^1^Department of Cell Biology, University of Alberta, Edmonton, Alberta T6G 2H7, Canada; ^2^Women and Children’s Health Research Institute, University of Alberta, Edmonton, Alberta T6G 2H7, Canada

**Keywords:** behavior, Cajal–Retzius cells, CXCL12, hippocampus, neurogenesis

## Abstract

The chemokine CXCL12 plays critical roles in the development of the hippocampus dentate gyrus during both embryogenesis and adulthood. While multiple cell types in the hippocampus express *Cxcl12*, their individual contributions to the dentate gyrus development and function remain unclear. Here, using *Cxcl12* reporter mice of both sexes, we characterize *Cxcl12* expression in Cajal–Retzius (CR) cells—neurons that guide dentate gyrus morphogenesis and influence hippocampal circuitry. We show that CR cells prominently express *Cxcl12* during early postnatal development, although both the number and proportion of *Cxcl12*-expressing CR cells decline significantly in adulthood. Notably, partial deletion of *Cxcl12* from hippocampal CR cells in male and female mice does not result in detectable changes in dentate gyrus architecture, adult neurogenesis, or specific behaviors. These findings suggest that CR cell-derived CXCL12 may be less critical for dentate gyrus development than previously assumed and underscore the complexity and potential redundancy of CXCL12 signaling in the hippocampus.

## Significance Statement

The chemokine CXCL12 regulates diverse aspects of hippocampal development, but the specific contributions of its various cellular sources remain unclear. Here, we show that Cajal–Retzius (CR) cells prominently express *Cxcl12* during early postnatal development, with the number of *Cxcl12*-expressing CR cells declining sharply in adulthood. Surprisingly, partial deletion of *Cxcl12* from CR cells does not impact dentate gyrus architecture, adult neurogenesis, or behavior. These findings suggest that CR cell-derived CXCL12 may not be essential for dentate gyrus development and point to potential redundancy in CXCL12 signaling within the hippocampus.

## Introduction

The chemokine CXCL12 and its receptors, CXCR4 and CXCR7, regulate diverse processes in central nervous system development, including neurogenesis, neurite outgrowth, neuronal migration, axon pathfinding, and synaptic plasticity (Li and Ransohoff, 2008; Mithal et al., 2012; Zhu and Murakami, 2012; Wu et al., 2017). In the hippocampus, granule cell progenitors in the dentate gyrus exhibit persistent expression of CXCR4 from early development through adulthood (Schultheiss et al., 2013). During embryogenesis, *Cxcr4* mRNA and protein are broadly distributed and can be detected in the dentate ventricular zone as early as embryonic day (E)13.5–E14.5 in mice, but their expression becomes progressively restricted. By birth, *Cxcr4* mRNA is largely confined to granule neuron progenitors within the dentate migratory stream and continues to decline postnatally, remaining prominent only in the neurogenic subgranular zone (Bagri et al., 2002; Lu et al., 2002; Tissir et al., 2004; Lopez-Bendito et al., 2008; Mimura-Yamamoto et al., 2017). Studies using CXCL12- or CXCR4-deficient mice, or receptor antagonists of CXCR4, such as AMD3100, have revealed precocious differentiation, delayed migration, disorganization of the granule cell layer, and ectopic distribution of granule cell progenitors in both embryonic and postnatal brains (Schultheiss et al., 2013; Mimura-Yamamoto et al., 2017). However, the precise cellular source of CXCL12 that promotes proper dentate gyrus development remains elusive. Early in situ mRNA hybridization studies in late embryonic (E17.5) to neonatal mice revealed strong *Cxcl12* expression in the hippocampal fissure region, which contains endothelial cells, leptomeninges cells, and Cajal–Retzius (CR) neurons (Bagri et al., 2002; Lu et al., 2002). These findings have been further supported by more recent single-cell RNA sequencing data (Hochgerner et al., 2018; Causeret et al., 2021). The complementary expression patterns of *Cxcl12* and *Cxcr4* in the late embryonic to neonatal hippocampus suggest the presence of a chemoattractive gradient that directs granule cell precursors toward the forming dentate gyrus (Bagri et al., 2002; Lu et al., 2002). Nonetheless, the individual contributions of these potential cellular sources of CXCL12 to dentate gyrus development and maintenance have yet to be fully elucidated.

CR cells are a class of earliest-born, transient neurons that populate the marginal zones of the developing neocortex and the hippocampal primordium (Martinez-Cerdeno and Noctor, 2014; Causeret et al., 2021; Elorriaga et al., 2023). They originate from four distinct regions of the telencephalon, with the cortical hem serving as the predominant source of CR cells destined for the neocortex and hippocampus (Forster et al., 2006; Yoshida et al., 2006; Louvi et al., 2007; Meyer et al., 2019; Ha et al., 2020; Vilchez-Acosta et al., 2022). CR cells from different origins exhibit distinct distribution properties, functional characteristics, and cell death dynamics. In the mouse neocortex, fewer than 5% of CR cells survive beyond the first postnatal week, whereas in the hippocampus, ∼20–30% persist throughout adulthood (Anstotz et al., 2016; Anstotz et al., 2018; van Bruggen et al., 2023). This differential persistence suggests that hippocampal CR cells may play crucial roles during postnatal development, necessitating their prolonged presence. Indeed, accumulating evidence indicates that surviving CR cells in the hippocampus influence local circuitry, behavior, and seizure susceptibility (Anstotz et al., 2022; Ramezanidoraki et al., 2023; Riva et al., 2023; Glaerum et al., 2024).

CR cells are best known for their production of reelin, an extracellular glycoprotein critical for cortical lamination (Ogawa et al., 1995; Vilchez-Acosta et al., 2022). However, the expression of other signaling molecules by CR cells and their potential roles in brain development remain largely unexplored. Notably, reelin deletion specifically from hippocampal CR cells only partially phenocopies the hippocampal morphogenesis defects observed following CR cell ablation, indicating that CR cell function in hippocampal development is only partly reelin-dependent (Elorriaga et al., 2025). This highlights the need to investigate other CR cell-derived factors that may contribute to hippocampal patterning and circuit assembly. Several studies in both embryonic and postnatal mouse brains have identified *Cxcl12* expression in CR cells in both the neocortex and hippocampus under physiological conditions (Bagri et al., 2002; Lu et al., 2002; Yamazaki et al., 2004; Causeret et al., 2021). Given that hippocampal CR cells contribute to the laminar organization of granule neurons and influence local circuitry (Anstotz et al., 2022; Vilchez-Acosta et al., 2022; Ramezanidoraki et al., 2023; Riva et al., 2023; Glaerum et al., 2024) and that CXCL12 signaling is essential for hippocampus development and homeostasis (Bagri et al., 2002; Lu et al., 2002; Mithal et al., 2012), it is important to define the specific role of CR cell-derived CXCL12 in hippocampus development and function. Here, we characterize the expression pattern of *Cxcl12* in the early postnatal and adult mouse hippocampus and investigate the functional consequences of its deletion specifically from hippocampal CR cells.

## Materials and Methods

### Mice

*ΔNp73-Cre* hemizygous mice (Tissir et al., 2009) were a generous gift from Dr. A. Pierani (Université de Paris), and the colony was maintained as hemizygous; only hemizygous Cre mice were used in this study. *Cxcl12^dsRED^*^/+^ knock-in mice (STOCK *Cxcl12^tm2.1Sjm^*/J, strain #022458, RRID:IMSR_JAX:022458; Ding and Morrison, 2013) and *Cxcl12^flox/flox^* mice [B6(FVB)-*Cxcl12^tm1.1Link^*/J, strain #021773, RRID:IMSR_JAX:021773; Greenbaum et al., 2013) were obtained from The Jackson Laboratory. All mice were group-housed on a 12/12 h day and night cycle with water and food *ad libitum*. Both male and female mice were used for experiments. All animal procedures were performed in accordance with the University of Alberta animal care committee’s regulations.

### Tissue preparations

Mice were intraperitoneally injected with sodium pentobarbital (Euthanyl, 240 mg/ml; Bimeda-MTC) and transcardially perfused with phosphate-buffered saline (PBS; Fisher Bioreagents, BP399-20), followed by 4% paraformaldehyde (PFA; Electron Microscopy Sciences, 19202). Brains were then extracted and postfixed overnight in 4% PFA at 4°C. Using a mouse brain matrix (Zivic Instruments; BSMYS005-1 for young mice, BSMAS005-1 for adult mice), brains were coronally trimmed, embedded in optimal cutting temperature (OCT) compound (Fisher HealthCare, 4585), frozen on dry ice, and stored at −80°C. Coronal sections were cut at 40 μm thickness using a cryostat (Leica Microsystems, CM1520), and stored at 4°C in PBS containing 0.02% sodium azide (BICCA, 7144.8-16). Alternatively, sections were mounted on Superfrost Plus microscope slides (Thermo Fisher Scientific, 12-550-5), air-dried overnight, and stored at −80°C.

### Immunofluorescence studies

For adult mice (>8 weeks old), three coronal brain sections per animal, spanning the dorsal dentate gyrus at the bregma −1.46 mm, −1.94 mm, and −2.46 mm, were selected for immunostaining. For younger mice, anatomically comparable sections were used.

Mounted section protocol: On Day 1, slides were postfixed in 10% phosphate-buffered formalin (Fisher Chemicals, SF100-4) for 10 min at room temperature, followed by three 5 min washes in PBS. If needed, heat-mediated antigen retrieval was performed by incubating slides in a citrate-based retrieval solution (Vector Laboratories, H-3300) at 95°C for 30 min in a water bath. After cooling to room temperature, slides were washed three times in PBS (5 min each) and then permeabilized with 0.3% Triton X-100 in PBS (PBST) for 20 min at room temperature. Slides were then blocked with 250 µl of 5% normal donkey serum (MilliporeSigma, D9663-10ML) in PBST for 20 min at room temperature. Primary antibodies, diluted in blocking solution, were applied, and slides were incubated overnight at 4°C in a humidified chamber. On Day 2, slides were washed three times in PBST (10 min each) and then incubated with fluorescent secondary antibodies diluted in blocking solution for 2 h at room temperature in the dark. Slides were subsequently washed twice in PBST and once in PBS (10 min each). To reduce background autofluorescence, we supplied Vector TrueVIEW autofluorescence quenching reagent (Vector Laboratories, SP8400) for 3 min, followed by a 10 min PBS wash. Nuclear counterstaining was performed using 5 μg/ml DAPI (Thermo Fisher Scientific, D3571) in PBS for 10 min at room temperature. Slides were washed three more times in PBS (5 min each), mounted with 75 µl of VectaShield Vibrance Antifade Mounting Medium (Vector Laboratories, H-1700-10), and coverslipped. After air-drying overnight, slides were sealed with clear nail polish and stored for imaging.

Free-floating section protocol: For free-floating immunofluorescence, the above steps were carried out in 24-well plates with three sections per well. Antigen retrieval, when required (e.g., for TRP73), was performed in a 95°C water bath for 10 min; however, note that the CXCL12 antibody is not compatible with antigen retrieval and was therefore processed without this step. After staining, sections were transferred onto Superfrost Plus microscope slides (Thermo Fisher Scientific, 12-550-5) immediately prior to mounting.

The following primary antibodies were used: rabbit anti-CXCL12 (PeproTech catalog #500-P87A, RRID:AB_148027, 1:500, not compatible with heat-mediated antigen retrieval), goat anti-dsRED (SICGEN catalog #AB8181-200, RRID:AB_2722750, 1:500), rabbit anti-TRP73 (Abcam catalog #ab40658, RRID:AB_776999, 1:500), mouse anti-RELN (MilliporeSigma catalog #MAB5364, RRID:AB_1293544, 1:500), mouse anti-DCX (Santa Cruz Biotechnologies catalog #sc-271390, RRID:AB_10610966, 1:25), rabbit anti-GFAP (DAKO catalog #Z033429-2, RRID:AB_10013382, 1:1,000), mouse anti-GFAP (MilliporeSigma catalog #G3893, RRID:AB_477010, 1:1,000), goat anti-SOX2 (R&D Systems catalog #AF2018, RRID:AB_355110, 1:500), rat anti-CTIP2 (Abcam catalog #ab18465, RRID:AB_2064130, 1:500), rabbit anti-PROX1 (Millipore catalog #AB5475, RRID:AB_177485, 1:500), rabbit anti-NFIA (MilliporeSigma catalog #HPA006111, RRID:AB_1854422, 1:250), rabbit anti-GABA (MilliporeSigma catalog #A2052, RRID:AB_477652, 1:1,000), mouse anti-GAD67 (MilliporeSigma catalog #MAB5406, RRID:AB_2278725, 1:500), and rabbit anti-Ki67 (Abcam catalog #ab15580, RRID:AB_443209, 1:1,000). The secondary antibodies used were donkey anti-goat Alexa Fluor 555 (Invitrogen catalog #A21432, RRID:AB_2535853, 1:1,000), donkey anti-rabbit Alexa Fluor 488 (Invitrogen catalog #A21206, RRID:AB_2535792, 1:1,000), and donkey anti-mouse Alexa Fluor 647 (Invitrogen catalog #A31571, RRID:AB_162542, 1:1,000).

### Protein extraction and immunoblotting

Hippocampi from Postnatal Day (P)10 mice were dissected, flash-frozen in liquid nitrogen, and stored at −80°C. The tissue from both hemispheres was homogenized in RIPA buffer (50 mM Tris–HCl, 150 mM NaCl, 0.1% Triton X-100, 0.1% SDS, 0.5% sodium deoxycholate, 1 mM EDTA), pH 7.4, supplemented with cOmplete Protease Inhibitors (Roche, 05892791001). Protein concentrations were determined using the Pierce BCA Protein Assay Kit (Thermo Fisher Scientific, 23227). Equal protein amounts were resolved on 4–15% Mini-PROTEAN TGX gels (Bio-Rad Laboratories, 4561086) and transferred to 0.45 µm nitrocellulose membranes (Amersham Protran, 10600002). Membranes were blocked with 2% BSA in Tris-buffered saline with 0.3% Tween 20 for 1 h and incubated overnight at 4°C with primary antibodies, followed by 1 h incubation with secondary antibodies at room temperature. Signals were detected using the Odyssey CLx (LI-COR) and quantified with Image Studio Lite v5.2. Primary antibodies used were rabbit anti-CXCL12 (PeproTech, catalog #500-P87A, RRID:AB_148027, 1:1,000), mouse anti-GAPDH (Advanced Immunochemical, catalog #2-RGM2, RRID:AB_2721282, 1:10,000), and mouse anti-vinculin (MilliporeSigma, catalog #V9131, RRID:AB_477629, 1:10,000). Secondary antibodies were goat anti-rabbit Alexa Fluor 680 (Thermo Fisher Scientific, catalog #A-21076, RRID:AB_2535736, 1:10,000) and goat anti-mouse DyLight 800 (Thermo Fisher Scientific, catalog #SA5-35521, RRID:AB_2556774, 1:10,000).

### Confocal microscopy and image analysis

Immunofluorescent images were taken using a laser-scanning confocal microscope (LSM 700, Carl Zeiss). For each mouse, three coronal brain slices spanning the dorsal dentate gyrus were processed for immunostaining, and a minimum of two of the six hemispheres were imaged for analysis. For each image, a tile scan was taken with a *z*-stack where thickness was kept consistent across animals in each group. The images were taken so that they included the hippocampal fissure and other hippocampus structures which were the region of interest for quantification purposes.

To quantify RELN^+^ CXCL12^+^ CR cells, we restricted the analysis to the hippocampal fissure and focused on cells exhibiting characteristic CR cell morphology—namely, a relatively small soma and a tadpole-like bipolar shape (Anstötz and Maccaferri, 2020)—to minimize inclusion of RELN^+^ interneurons present in the same region.

To quantify the density of GABAergic neurons and astrocytes, we obtained single *z*-stack images with identical optical thickness parameters across groups. These images were centered on one of three defined regions of interest: the CA1 region, the hippocampal fissure, or the hilus between the suprapyramidal and infrapyramidal blades of the dentate gyrus. Laser power and acquisition settings were maintained constant across all groups.

Cell counting and area measurements were performed using Fiji ImageJ software (v1.53). The hippocampal fissure area was defined as 60 µm above and below the hippocampal fissure (Pahle et al., 2020). The subgranular zone is defined as a layer of cells expanding 5 μm into the hilus and 15 μm into the granular cell layer (Hourigan et al., 2021). To measure the width of the CTIP2-positive cornu ammonis (CA) regions, a single line was drawn and measured along the CA regions: proximal, medial, and distal. To measure the PROX1^+^ and CTIP2^+^ cell density within the dentate gyrus, the dentate gyrus area was defined and measured; a threshold and watershed binary were applied before using Fiji ImageJ Particle Analysis to count the number of cells 4–500 µm^2^ in area. Cell density was normalized to the defined areas of interest, with two to three brain sections analyzed per animal. To account for the nested structure of the data when comparing genotypes, we performed nested one-way ANOVA tests. Brain sections were treated as nested observations within each animal to capture intra-animal variability and ensure that statistical comparisons reflected differences at the animal level rather than at the level of individual sections. Nested one-way ANOVA was conducted in GraphPad Prism, with genotype as the main factor and brain sections nested within animals. Statistical analyses are detailed in Extended Data Figure 1-1.

### Behavior tests

At least 1 h before each behavioral experiment, mice were transported from their housing area to the testing area to acclimate to a white noise machine, which played continuously at ∼60 dB during all behavior tests. Each behavior test was conducted on a separate day. All mouse behaviors were recorded, tracked, and analyzed using EthoVision 17 (Noldus). The tracking was based on the mouse's center point as detected by EthoVision 17, with the software also defining nose and tail points. After testing, the mice were returned to their home cages, and the arena was thoroughly cleaned with 70% ethanol before testing the next mouse.

#### Open-field assay

Mice were placed in a white arena measuring 40 × 40 cm. Their behavior and movement were recorded and tracked over a 15 min period. The center zone was defined as a 30 × 30 cm area in the middle of the arena.

#### Light/dark box

The light/dark box was divided into two sections: a 25 × 40 cm open section with transparent acrylic walls and a 17.5 × 40 cm dark section with black acrylic walls and a black plastic cover. A 5-cm-wide opening connected the light-exposed and dark sections. Mice were placed in the light section and could freely move between the light and dark areas for 10 min.

#### Elevated zero maze

The elevated zero maze had a total diameter of 50 cm and a track width of 5 cm, divided into four equal sections. Two sections were enclosed by 20 cm tall acrylic walls (closed sections), while the other two sections were open (open sections). The maze was elevated 61 cm above the ground. Mice were placed in the maze and could move freely between the sections. Their behavior and movement were recorded and tracked for 10 min.

#### Spontaneous Y maze

Mice were placed in the center of a white Y-shaped arena, with each arm measuring 35 × 5 cm. Mice could move freely around the arena and were tracked for 8 min. An alternation was defined as the number of times a mouse visited unique arms sequentially without revisiting a previous arm (e.g., moving from Arm A to B to C counted as one alternation but moving from Arm A to B to A did not). The alternation index was calculated by dividing the number of alternations by the maximum possible alternations, as determined by EthoVision 17.

#### Fear conditioning

Fear conditioning was conducted using the Ugo Basile 46000 Fear Conditioning System, controlled by EthoVision 17. On the first day, mice were placed in the arena for 3 min and then trained with two tone-shock pairings, each separated by 80 s intervals. A 2 kHz tone played for 20 s, and a 0.5 mA footshock was delivered during the last 2 s. On the second day, mice were returned to the arena for 5 min, and the time they spent immobile (freezing) was tracked to assess context-dependent memory. On the third day, the arena walls were covered with checkmark sheets, the electric grid was covered with a plastic tile, and a cup with vanilla essence was placed in the chamber. Mice could move freely in the arena. Mice were placed in the arena for 3 min followed by two 20 s tones played at 80 s intervals without a footshock. The time spent freezing in response to the tone was tracked to assess cue-dependent memory.

## Results

### *Cxcl12* is expressed by CR cells in the postnatal hippocampus

**Figure 1.**
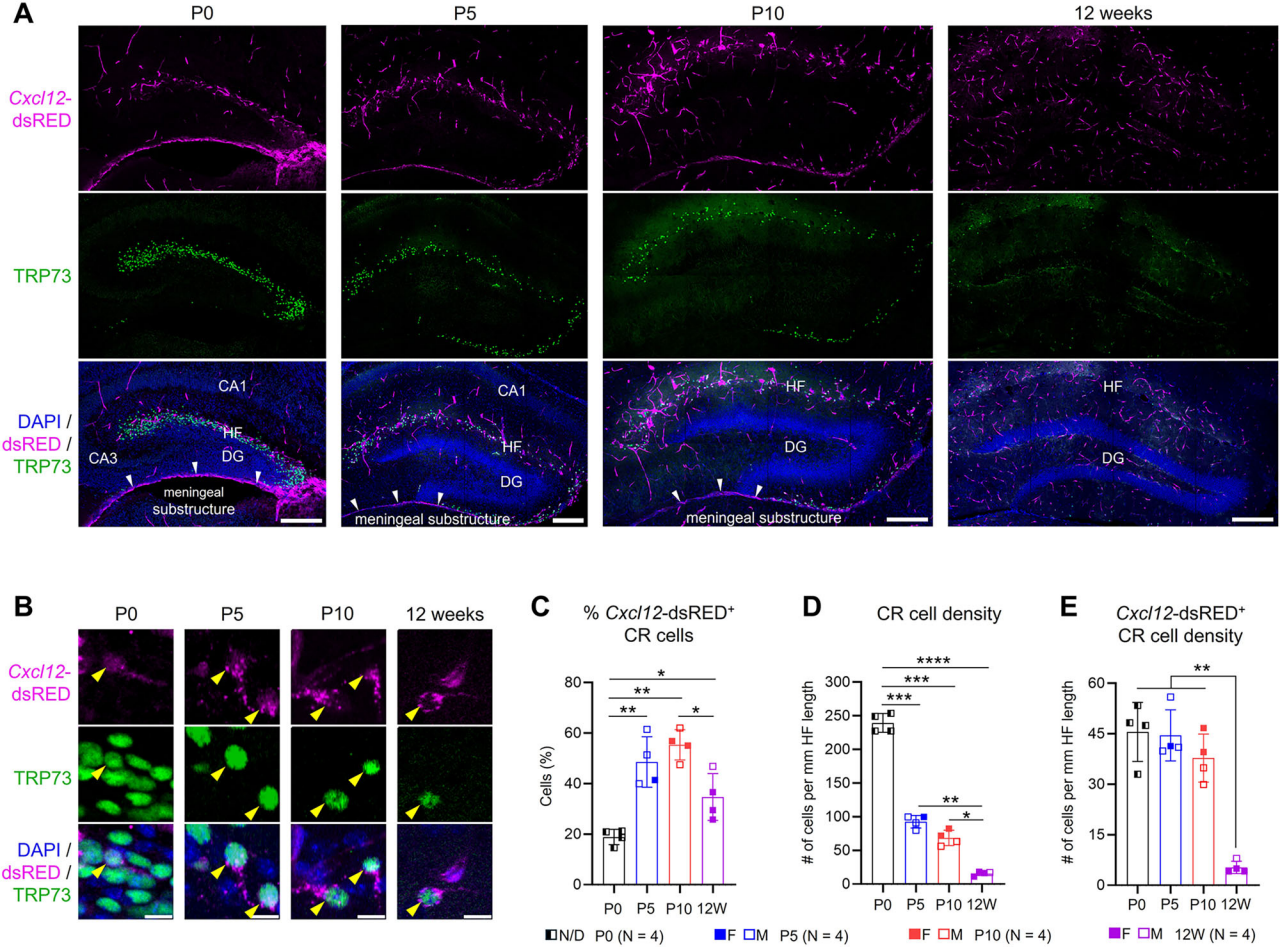
*Cxcl12* is expressed by CR cells in the postnatal hippocampus. ***A***, Representative confocal images showing coimmunostaining for dsRED and CR cell marker TRP73 in the hippocampus at P0, P5, P10, and 12 weeks. White arrowheads indicate the meningeal substructure ventral to the hippocampus. CA1, cornu ammonis 1; CA3, cornu ammonis 3; HF, hippocampal fissure; DG, dentate gyrus. Scale bars, 200 µm. ***B***, High-magnification images showing colocalization of dsRED and TRP73 in CR cells (yellow arrowheads). Scale bars, 5 µm. ***C–E***, Quantification of (***C***) the percentage of dsRED-expressing CR cells, (***D***) total CR cell density, and (***E***) dsRED-expressing CR cell density at P0, P5, P10, and 12 weeks in the hippocampal fissure area. Data are presented as scatterplots with individual data points (each representing one animal), and error bars denote ±SD. Three sections were analyzed per animal. Solid and open symbols represent female and male mice, respectively; half-solid symbols represent P0 mice with undetermined sex. Statistical analyses were performed using nested one-way ANOVA followed by Tukey's post hoc test. *p < 0.05; **p < 0.01; ***p < 0.001; **p < 0.0001. Extended Data Figures 1-1 and 1-2 support Figure 1.

**Figure 2.**
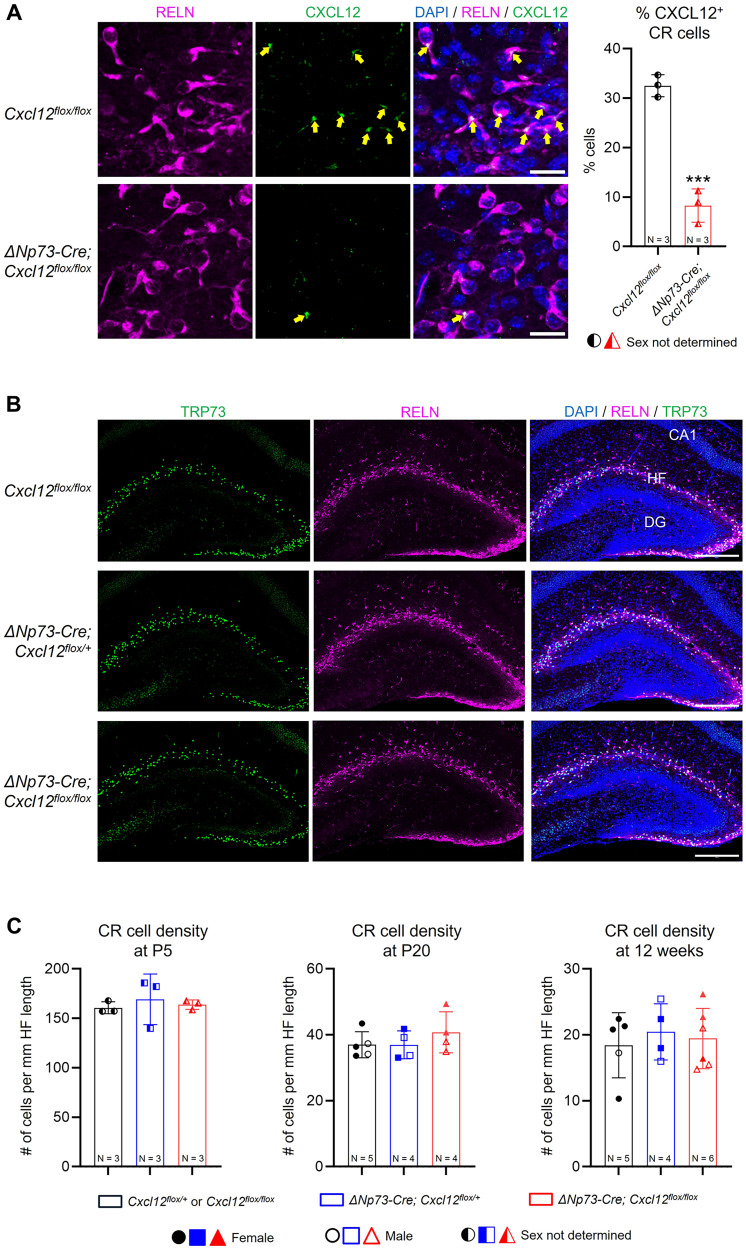
Partial deletion of CXCL12 in CR cells does not affect their survival in the postnatal hippocampus. ***A***, Representative confocal images showing coimmunostaining of CXCL12 and the CR cell marker reelin (RELN) in P5 control (*Cxcl12^flox/flox^*) and knock-out (Δ*Np73Cre; Cxcl12^flox/flox^*) hippocampus. Arrows indicate double-positive cells. Scale bars, 20 µm. Quantification of the percentage of CXCL12-positive CR cells is shown at right. ***B***, Representative confocal images showing coimmunostaining of CR cell markers RELN and TRP73 at P20. CA1, cornu ammonis 1; HF, hippocampal fissure; DG, dentate gyrus. Scale bars, 200 µm. ***C***, Quantifications of RELN^+^ TRP73^+^ CR cell density in the hippocampal fissure at P5, P20, and 12 weeks. Data are shown as scatterplots with individual data points (each representing one animal); error bars indicate ±SD. Three sections were analyzed per animal. Solid and open symbols represent female and male mice, respectively; half-solid symbols represent P5 mice with undetermined sex. Statistical analyses were performed using nested *t* test or nested one-way ANOVA with Tukey's post hoc test. ***p < 0.001. Extended Data Figure 2-1 supports Figure 2.

Although both *Cxcl12* and *Cxcr4* are detected as early as E13.5–E14.5, their complementary expression patterns become particularly striking in the dentate gyrus between E17.5 and birth, coinciding with the progressive restriction of *Cxcr4* expression (Bagri et al., 2002; Lu et al., 2002; Lopez-Bendito et al., 2008). We therefore sought to identify the specific cellular sources of *Cxcl12* around the time of birth. To accurately map *Cxcl12*-expressing cells, we utilized the *Cxcl12^dsRED/+^* knock-in mice, in which the fluorescence reporter *dsRED* is inserted into the endogenous *Cxcl12* locus (Ding and Morrison, 2013). The reporter dsRED serves as an indicator of endogenous *Cxcl12* promoter activity, thereby reflecting active *Cxcl12* expression. At P0, *Cxcl12*–dsRED expression was observed along the hippocampal fissure (Fig. 1*A*). Colocalization with the CR cell-specific marker TRP73 revealed that ∼20% of CR cells express *Cxcl12*–dsRED at P0 (Fig. 1*B*,*C*). Additionally, *Cxcl12*–dsRED was detected in a meningeal substructure ventral to the hippocampus (Mercier and Arikawa-Hirasawa, 2012; Bifari et al., 2017) as well as the vasculatures (Fig. 1*A*; Extend Data Fig. 1-2), consistent with previous single-cell RNA data, as well as a study using the same reporter line (Abe et al., 2018). At P5 and P10, the expression patterns of *Cxcl12*–dsRED remained similar to that at P0 (Fig. 1*A*; Extend Data Fig. 1-2); however, the proportion of *Cxcl12*–dsRED-expressing CR cells increased to ∼50% for both time points (Fig. 1*B*,*C*). In the adult (12-week-old) hippocampus, *Cxcl12*–dsRED was observed in the vasculature (Extend Data Fig. 1-2) and in ∼35% of CR cells (Fig. 1*A*–*C*). In the hippocampal fissure region, CR cell density decreases drastically from early postnatal weeks to adults (Fig. 1*D*), mainly due to programmed cell death, although the growth of the developing structure and resulting cellular dilution might also play a role (Anstotz et al., 2016; van Bruggen et al., 2023). We found that the density of *Cxcl12*–dsRED-expressing CR cells was similar among P0, P5, and P10 hippocampi but decreased significantly by 12 weeks of age (Fig. 1*E*). Altogether, our data demonstrate that hippocampal CR cells are a prominent cellular source of *Cxcl12* expression during the first 2 postnatal weeks. Notably, *Cxcl12*-expressing CR cells persist longer than their *Cxcl12*-negative counterparts, undergoing cell death only after P10.

10.1523/ENEURO.0245-25.2025.f1-1 Figure 1-1 **Statistical summary table.** Detailed statistical information, including the type of test performed, statistical values, and significance levels, for all analyses. Download Figure 1-1, XLS file.

10.1523/ENEURO.0245-25.2025.f1-2 Figure 1-2 ***Cxcl12* expression in Cajal-Retzius cells and the vasculature in the postnatal hippocampus.** Representative confocal images showing co-immunostaining for dsRED and Cajal-Retzius (CR) cell marker TRP73 in the hippocampus at postnatal day (P) 0, P5, P10, and 12 weeks. Yellow arrowheads indicate the vasculature. White arrows indicate TRP73^+^ CR cells. Scale bars, 25 µm. Download Figure 1-2, TIF file.

### Partial deletion of CXCL12 in CR cells does not affect their survival in the postnatal hippocampus

Our reporter line study suggests that CXCL12 derived from CR cells may contribute to early hippocampal development, particularly given their preferential survival before P10 (Fig. 1*C*,*E*). To selectively delete *Cxcl12* from CR cells, we generated *ΔNp73-Cre; Cxcl12^flox/flox^* conditional knock-out mice. The *ΔNp73-Cre* line drives Cre recombinase expression specifically in cortical hem–derived CR cells, which constitute the majority of hippocampal CR cells (Tissir et al., 2009; van Bruggen et al., 2023). We first assessed the extent of CXCL12 deletion. Although immunoblotting revealed no change in overall CXCL12 protein levels in whole hippocampal lysates at P10 (Extend Data Fig. 2-1), immunostaining for CXCL12 and the CR cell marker reelin (RELN) at P5 demonstrated a marked reduction of CXCL12 in CR cells. In *Cxcl12^flox/flox^* control mice, ∼32% of CR cells exhibited CXCL12 immunoreactivity, appearing as punctate labeling in the soma and diffuse signal along dendrites, whereas only ∼8% of CR cells in *ΔNp73-Cre; Cxcl12^flox/flox^* mice showed detectable CXCL12 staining, indicating a partial yet significant loss of CXCL12 expression in CR cells (Fig. 2*A*). Given that meningeal-derived CXCL12 regulates CR cell migration and positioning in the developing neocortex (Borrell and Marin, 2006), we asked whether CR cell-derived CXCL12 might play a similar but cell-autonomous role in the hippocampus. However, CR cells in knock-out mice displayed comparable localization and density along the hippocampal fissure of the dentate gyrus at P5, P20, and 12 weeks of age (Fig. 2*B,C*). As hippocampal CR cells undergo extensive programmed cell death between P5 and P20 (Anstotz et al., 2016; van Bruggen et al., 2023), our findings suggest that partial loss of CXCL12 does not affect their postnatal survival.

10.1523/ENEURO.0245-25.2025.f2-1 Figure 2-1 **Partial deletion of CXCL12 in Cajal-Retzius cells does not alter hippocampal CXCL12 protein levels.** Immunoblot showing CXCL12 protein in whole hippocampal lysates from postnatal day (P) 10 control and *ΔNp73-Cre; Cxcl12^flox/flox^* mice. Vinculin (VCL) and GAPDH serve as loading controls. Quantification of CXCL12 levels normalized to loading controls is shown at right. Download Figure 2-1, TIF file.

### Partial deletion of CXCL12 in CR cells does not disrupt early postnatal hippocampal morphogenesis or neurogenesis

**Figure 3.**
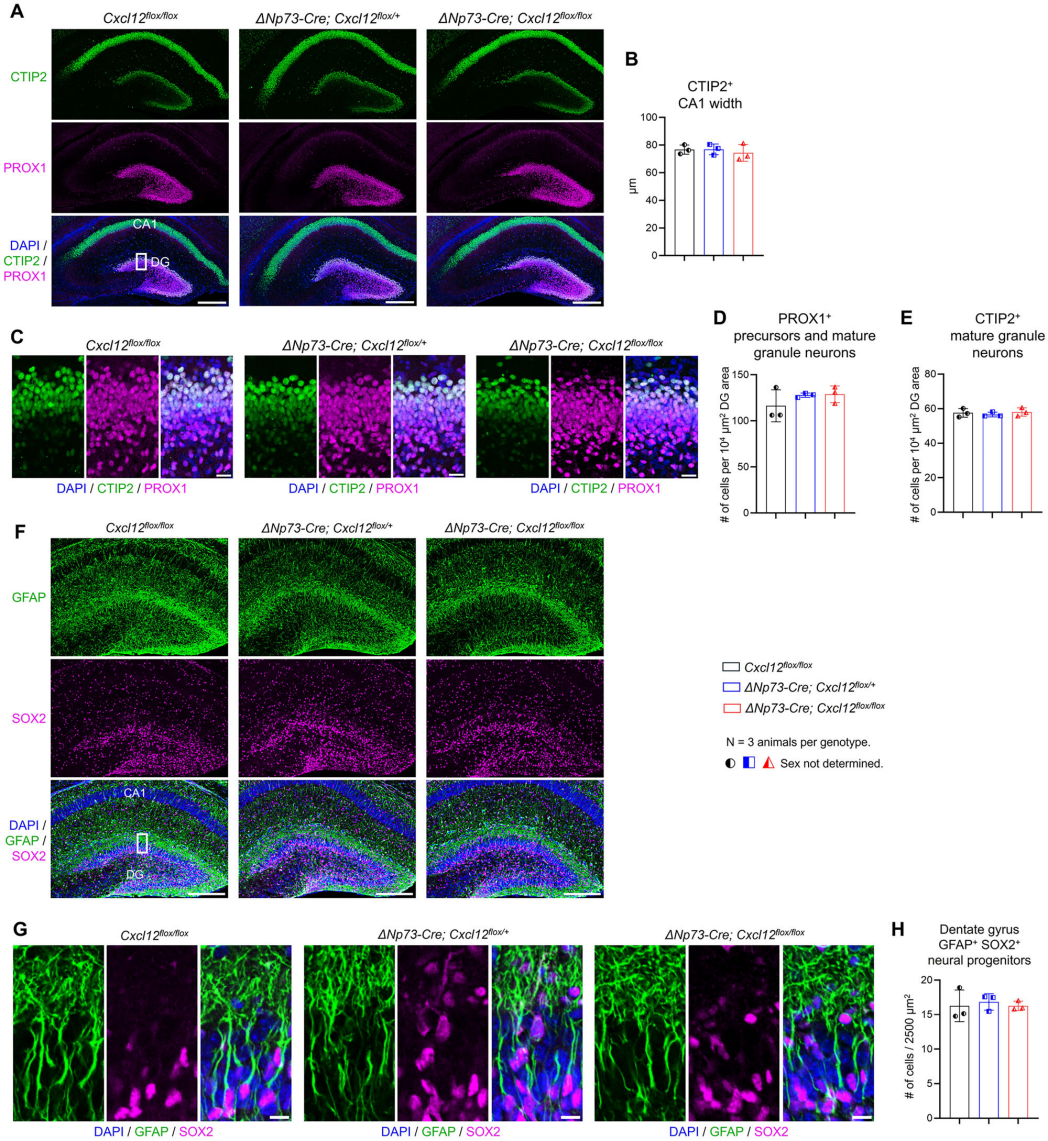
Partial deletion of CXCL12 in CR cells does not disrupt early postnatal hippocampal morphogenesis or neurogenesis. ***A***, Representative confocal images showing coimmunostaining of CTIP2 and PROX1 in the P5 hippocampus. CTIP2 labels CA1 pyramidal neurons and mature dentate gyrus (DG) granule neurons. PROX1 labels granule neuron precursors and mature granule neurons. CA1, cornu ammonis 1. The boxed region indicates the DG area shown in ***C***. Scale bars, 200 µm. ***B***, Quantification of CA1 width based on CTIP2 immunostaining. ***C***, High-magnification images of CTIP2 and PROX1 coimmunostaining in the DG. Scale bar, 20 µm. ***D, E***, Quantification of PROX1^+^ (***D***) and CTIP2^+^ (***E***) granule neuron densities in the DG. ***F***, Representative overview images of GFAP and SOX2 coimmunostaining in the P5 hippocampus. The boxed region indicates the DG area shown in ***G***. Scale bars, 200 µm. ***G***, High-magnification images of GFAP and SOX2 coimmunostaining in the DG. Scale bar, 10 µm. ***H***, Quantification of GFAP^+^ SOX2^+^ progenitor cell density in the subgranular zone. Data are presented as scatterplots, with each data point representing one animal. Error bars indicate ±SD. Three sections were analyzed per animal. Half-solid symbols represent P5 mice with undetermined sex. Statistical analyses were performed using nested one-way ANOVA followed by Tukey’s post hoc test.

Dentate gyrus morphogenesis primarily occurs within the first 10 postnatal days in mice (Altman and Bayer, 1990; Yu et al., 2014; Hochgerner et al., 2018). The CXCL12–CXCR4 axis plays a crucial role in this process, as evidenced by the absence of a properly developed dentate gyrus in mice lacking CXCR4 (Lu et al., 2002). Although CR cells have long been hypothesized as a significant source of CXCL12, which promotes the migration of granule precursor cells from the neuroepithelium to the dentate gyrus granular layer (Bagri et al., 2002; Lu et al., 2002; Hatami et al., 2018), this has not been formally tested. To address this gap, we utilized our Δ*Np73-Cre; Cxcl12^flox/flox^* knock-out mice to investigate whether partial deletion of CXCL12 from CR cells affects early postnatal hippocampal development. At P5, the overall morphology of the hippocampus was indistinguishable between Δ*Np73-Cre; Cxcl12^flox/flox^* knock-out and control mice (Fig. 3*A*). The width of the CA1 region, labeled by CTIP2, was comparable across all groups of animals (Fig. 3*B*). Focusing on dentate gyrus granule neurons, we used the pan-granule neuron marker PROX1 (Karalay et al., 2011) and observed that the total number of granule neuron precursors and mature granule neurons was similar between control and knock-out mice (Fig. 3*A*,*C*,*D*). Additionally, the number of CTIP2^+^ postmitotic granule neurons (Simon et al., 2012) remained unchanged in the knock-out mice (Fig. 3*A*,*C*,*E*). When examining progenitors in the subgranular zone, identified by their coexpression of GFAP and SOX2 along with their thick primary radial processes, we found that the number of progenitors was comparable across all groups of mice (Fig. 3*F*–*H*).

**Figure 4.**
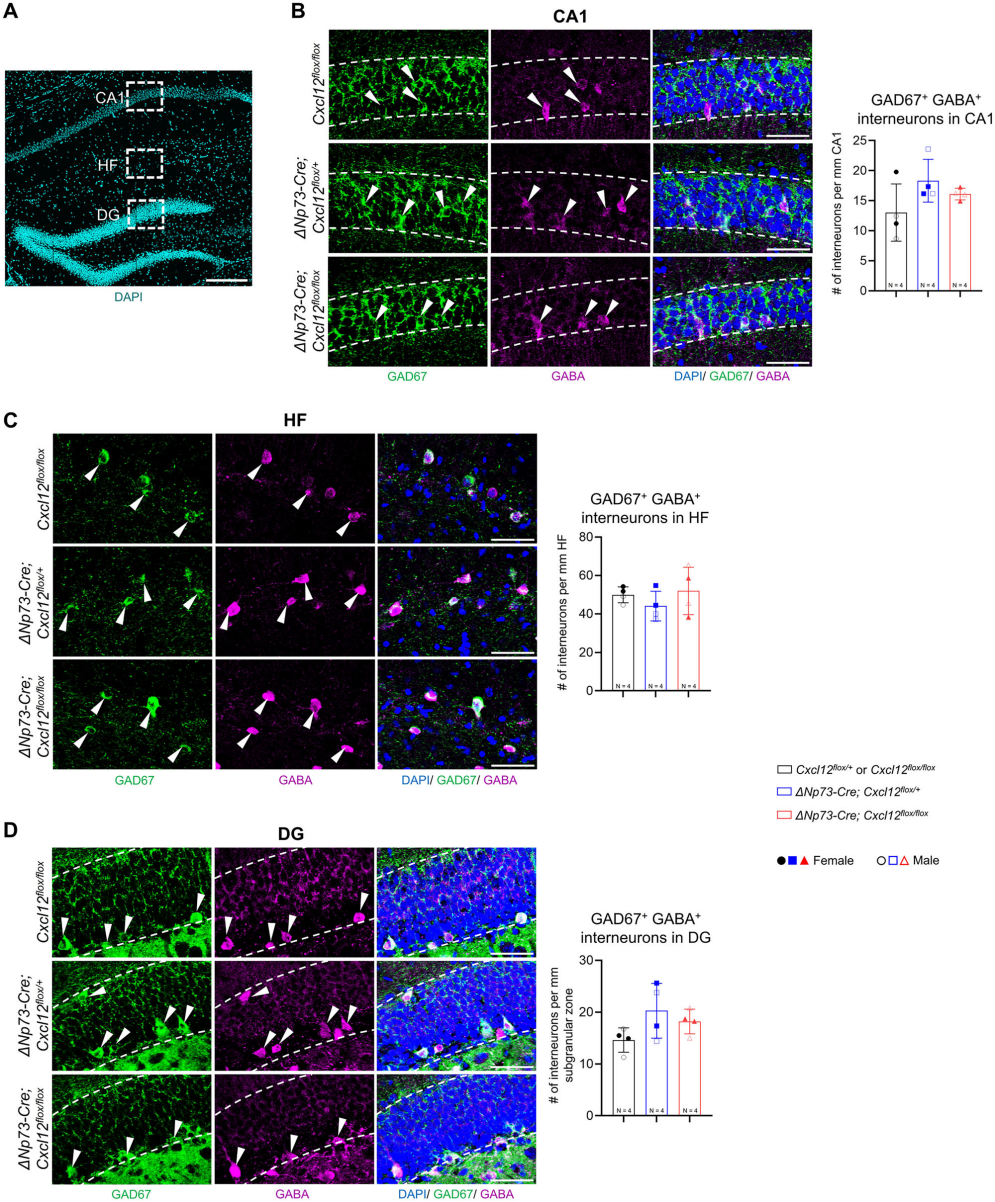
Interneuron density in the late postnatal hippocampus is unchanged following partial deletion of CXCL12 from CR cells. ***A***, Representative DAPI-stained hippocampal section showing the regions analyzed for interneuron quantification. Scale bar, 100 µm. ***B–D***, Representative confocal images showing coimmunostaining for GAD67 and GABA in the CA1 pyramidal layer (***B***), hippocampal fissure (HF; ***C***), and dentate gyrus (DG) granule cell layer (***D***) at P20. Dashed lines indicate the quantified regions, and arrowheads mark GAD67^+^ GABA^+^ double-positive interneurons. Scale bar, 50 µm. Quantification of total GAD67^+^ GABA^+^ interneurons is shown at the right. Error bars indicate mean ± SD. Two sections were analyzed per animal; solid and open symbols represent female and male mice, respectively. Statistical analyses were performed using nested one-way ANOVA with Tukey's post hoc test.

**Figure 5.**
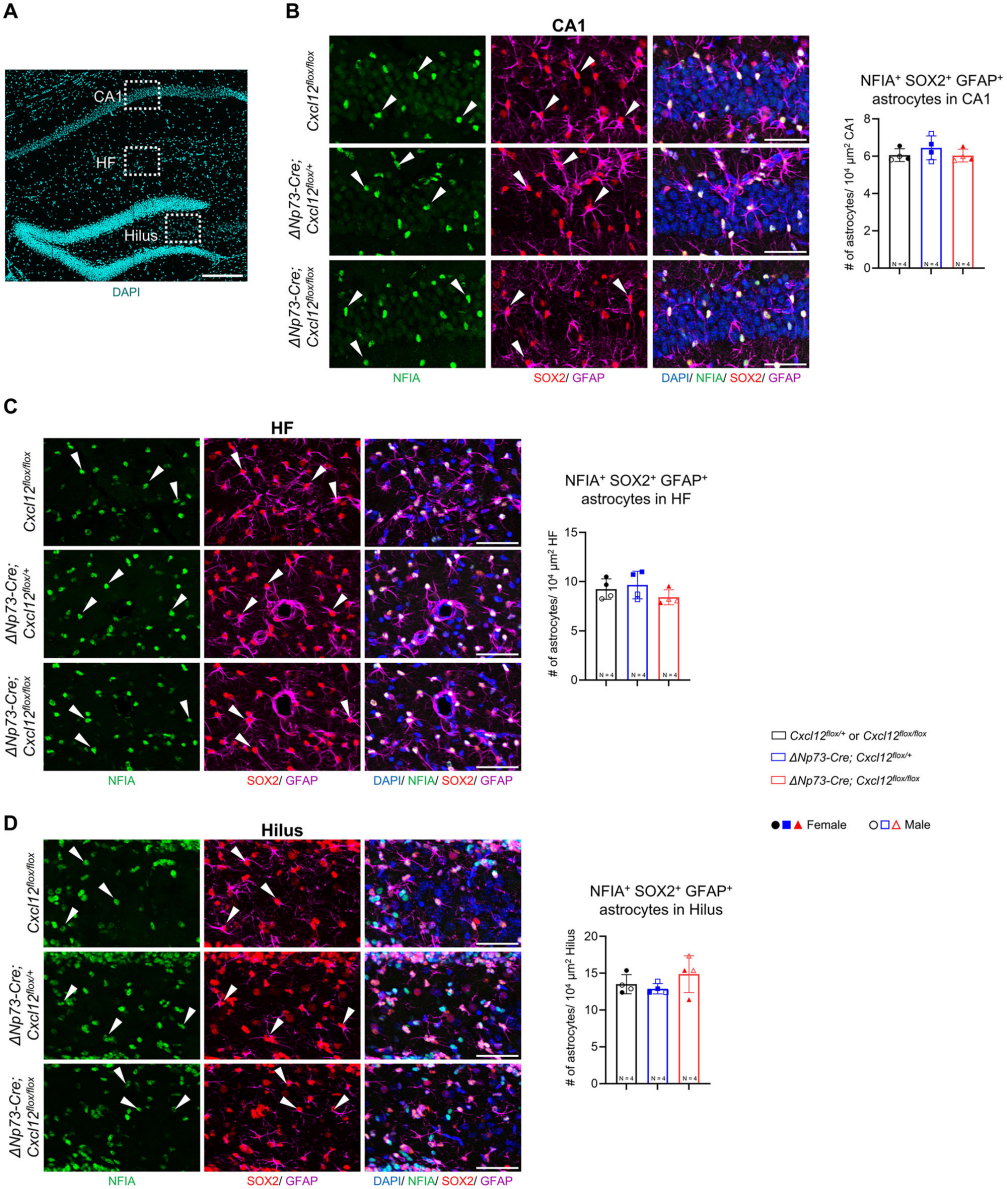
Selective loss of CXCL12 in CR cells does not impact astrocyte density in the postnatal hippocampus. ***A***, Representative DAPI-stained hippocampal section showing the regions analyzed for astrocyte quantification. Scale bar, 100 µm. ***B–D***, Representative confocal images showing coimmunostaining for NFIA, GFAP, and SOX2 in the CA1 pyramidal layer (***B***), hippocampal fissure (HF; ***C***), and dentate gyrus (DG) granule cell layer (***D***) at P20. White arrowheads indicate NFIA^+^ GFAP^+^ SOX2^+^ astrocytes. Scale bar, 50 µm. Quantification of total NFIA^+^ GFAP^+^ SOX2^+^ astrocytes is shown at the right. Error bars indicate mean ± SD. Two sections were analyzed per animal; solid and open symbols represent female and male mice, respectively. Statistical analyses were performed using nested one-way ANOVA with Tukey's post hoc test.

Beyond granule neurons and their progenitors, CXCL12 signaling has also been implicated in interneuron development and astrocyte function. Deletion of the CXCL12 receptor CXCR4 from GABAergic interneurons impairs interneuron migration into the hippocampus during embryogenesis (Li et al., 2008), while CXCR4 mediates glutamate exocytosis from astrocytes (Cali and Bezzi, 2010). To determine whether these populations were affected by partial loss of CXCL12 from CR cells, we examined P20 hippocampi by immunostaining for GABAergic neurons using GAD67 and GABA and for astrocytes using NFIA, GFAP, and SOX2. The density of GABAergic neurons did not differ significantly between knock-out and control mice across the CA1 pyramidal layer, hippocampal fissure, or the dentate gyrus granule cell layer (Fig. 4). Similarly, astrocyte density within the CA1 region, hippocampal fissure, and hilus was unaffected by partial loss of CXCL12 from CR cells (Fig. 5).

Overall, these findings indicate that partial deletion of CXCL12 from CR cells does not significantly affect dentate gyrus morphogenesis or alter interneuron or astrocyte populations during early and late postnatal hippocampal development.

### Partial deletion of CXCL12 in CR cells preserves adult dentate gyrus neurogenesis

**Figure 6.**
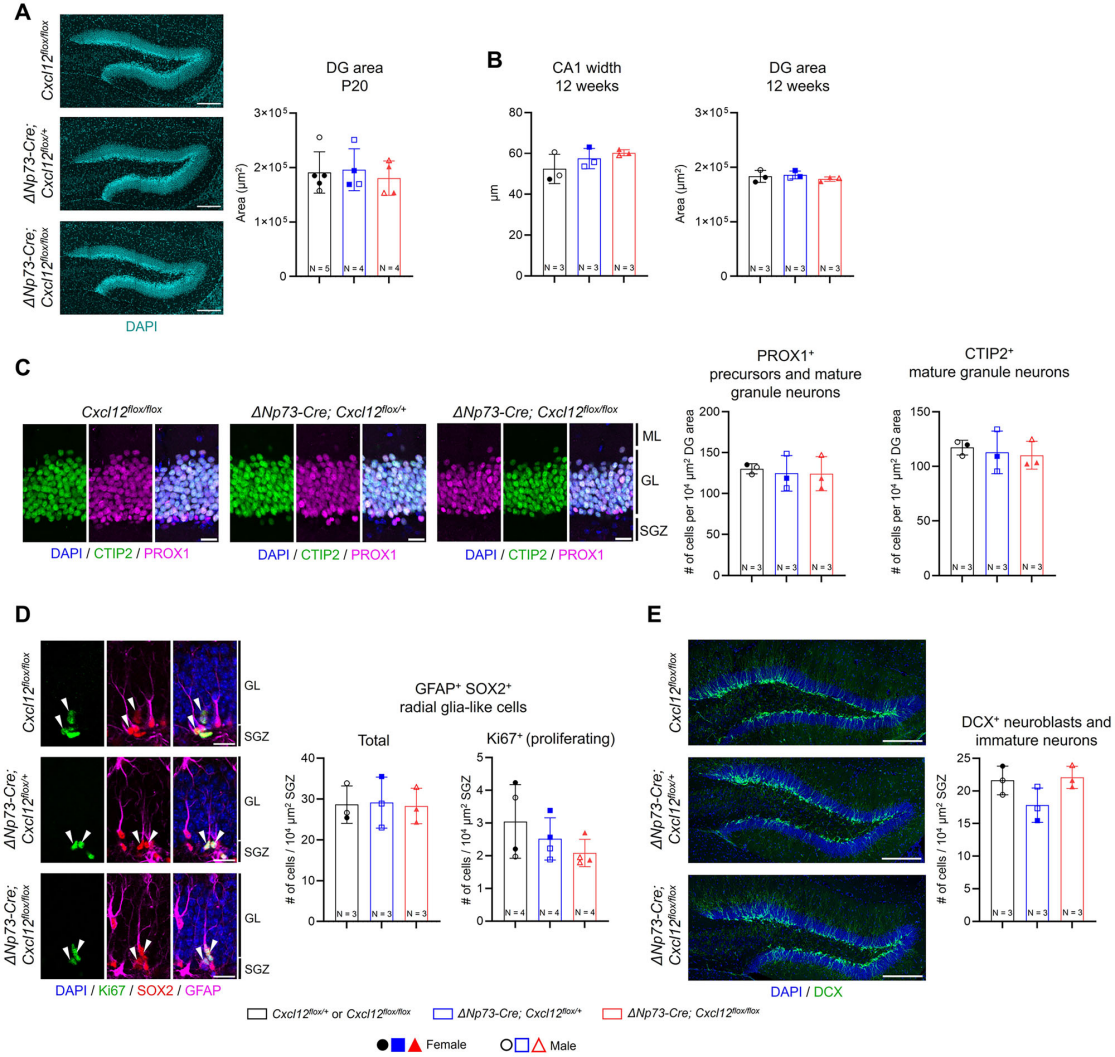
Partial deletion of CXCL12 in CR cells preserves adult dentate gyrus neurogenesis. ***A***, Representative DAPI staining showing the overall morphology of the dentate gyrus (DG) at P20. Quantification of the DG area is shown at the right. Scale bars, 200 µm. ***B***, Quantifications of CA1 width based on CTIP2 immunostaining and DG area based on DAPI staining in 12-week-old mice. ***C***, Representative high-magnification images of CTIP2 and PROX1 coimmunostaining in the 12-week-old control DG. Quantification of PROX1^+^ and CTIP2^+^ granule neuron densities is shown at right. Scale bar, 20 µm. SGZ, subgranular zone; GL, granular layer; ML, molecular layer. ***D***, Representative confocal images showing coimmunostaining for GFAP, SOX2, and Ki67 in the DG of 12-week-old mice. White arrowheads indicate proliferating (Ki67^+^) GFAP^+^ SOX2^+^ radial glia-like cells. Scale bar, 20 µm. Quantification of total and proliferating (Ki67^+^) GFAP^+^ SOX2^+^ radial glia-like cells is shown at the right. ***E***, Representative low-magnification images of DCX immunostaining in the DG. Scale bar, 200 µm. Quantification of DCX^+^ neuroblasts and immature neurons is shown at the right. Data are presented as scatterplots, with each data point representing one animal. Error bars indicate ±SD. Three sections were analyzed per animal. Solid and open symbols represent female and male mice, respectively. Statistical analyses were performed using nested one-way ANOVA with Tukey's post hoc test.

The adult dentate gyrus is one of two brain regions where neurogenesis persists under physiological conditions (Kempermann et al., 2015). During this process, CXCR4 and CXCR7 are expressed by neural progenitors and immature neurons (Schultheiss et al., 2013; Banisadr et al., 2016), and the CXCL12–CXCR4/CXCR7 signaling axis plays a key role in maintaining the neural stem cell pool, facilitating neuronal differentiation, and guiding the integration of new neurons into the dentate gyrus (Schultheiss et al., 2013; Abe et al., 2018; Trousse et al., 2019). Given the importance of CXCL12 in these processes, we asked whether its partial deletion specifically from CR cells would affect adult neurogenesis. Since the transition from developmental to adult neurogenesis occurs around P20 (Gilley et al., 2011; Hochgerner et al., 2018), we examined the dentate gyrus at this age. Gross morphology and overall size of the dentate gyrus were similar between groups (Fig. 6*A*). Analysis of the cytoarchitecture of the adult hippocampus in 12-week-old mice revealed no differences among groups (Fig. 6*B*). Marker analysis showed comparable numbers of PROX1^+^ and CTIP2^+^ granule neurons in control and knock-out mice (Fig. 6*C*). We extended our analysis to radial glia-like cells, the adult neural stem cells located in the subgranular zone, which are characterized by GFAP and SOX2 coexpression and the presence of a prominent radial process. Both total and proliferating (Ki67^+^) radial glia-like cell numbers were similar across genotypes (Fig. 6*D*). Although a trend toward lower numbers of Ki67^+^ cells in animals with progressive genetic deletion of *Cxcl12* was observed, the effect size was small and variability overlapped between genotypes. Such subtle effects, if present, may require larger cohorts to detect. Furthermore, adult-born neuroblasts and immature neurons labeled with DCX displayed typical morphology and localization, with similar numbers observed across genotypes (Fig. 6*E*). Overall, these findings indicate that partial deletion of CXCL12 from CR cells does not significantly impact adult neurogenesis in the dentate gyrus.

### Partial deletion of CXCL12 in CR cells does not alter hippocampal-dependent behaviors in adult mice

**Figure 7.**
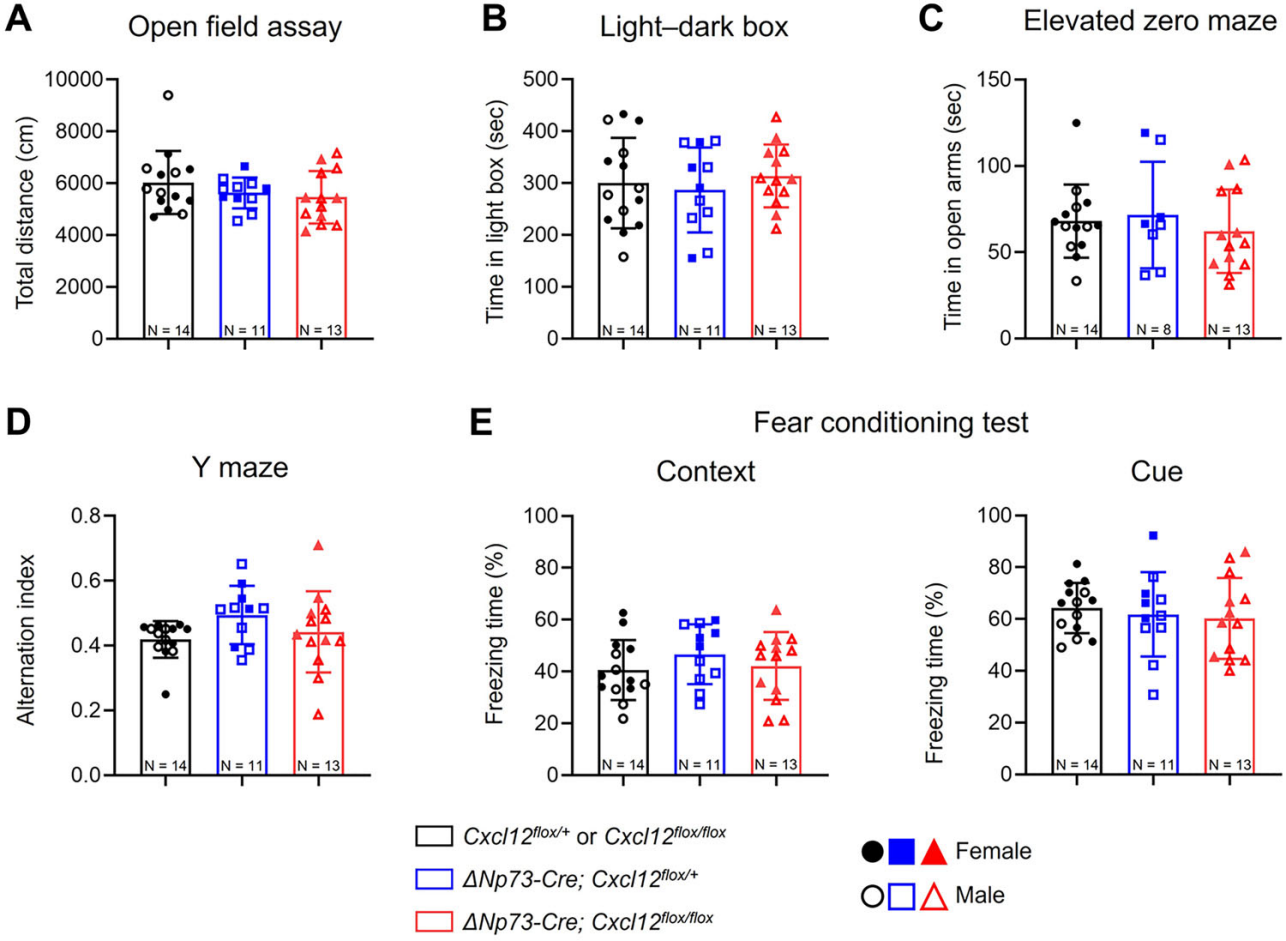
Partial deletion of CXCL12 in CR cells does not alter hippocampal-dependent behaviors in mice. Adult (12–14-week-old) mice were subject to (***A***) the open-field assay, (***B***) the light/dark box assay, (***C***) the elevated zero maze test, (***D***) the spontaneous Y maze test, and (***E***) the context and cued fear conditioning tests. Data are presented as scatter plots with all data points shown and error bars representing ±SD. Each data point (*N*) is an individual animal. Solid and open symbols represent data from female and male mice, respectively. Statistical analyses were performed using ordinary one-way ANOVA with Tukey's post hoc test. Extended Data Figure 7-1 supports Figure 7.

CR cells contribute to hippocampal-dependent learning and memory (Anstotz et al., 2022; Riva et al., 2023), and disruption of the CXCL12–CXCR4/CXCR7 signaling axis has been associated with cognitive deficits (Kolodziej et al., 2008; Trousse et al., 2019). To determine whether partial deletion of CXCL12 from CR cells affects adult behavior, we assessed a range of hippocampal-dependent tasks. Although our histological analyses revealed no major structural alterations, we hypothesized that subtle circuit-level changes could still impact function. In the open-field test, knock-out mice exhibited normal locomotor activity, as indicated by similar distances traveled compared with controls (Fig. 7*A*; Extended Data Fig. 7-1*A*). Anxiety-like behavior, measured via the light/dark box and elevated zero maze, also remained unchanged: knock-out mice showed a similar preference for the light compartment (Fig. 7*B*; Extended Data Fig. 7-1*B*) and spent comparable time in the open arms of the maze (Fig. 7*C*; Extended Data Fig. 7-1*C*). Spatial working memory was assessed using the spontaneous Y maze test, which revealed no differences in the alternation index between groups (Fig. 7*D*; Extended Data Fig. 7-1*D*). Finally, both context- and cue-dependent fear conditioning assays showed comparable freezing behavior in knock-out and control mice (Fig. 7*E*; Extended Data Fig. 7-1*E*,*F*), indicating intact fear memory. Collectively, these results suggest that partial deletion of CXCL12 from CR cells does not lead to measurable deficits in hippocampal-dependent behaviors under standard testing conditions.

10.1523/ENEURO.0245-25.2025.f7-1 Figure 7-1 **Partial deletion of CXCL12 in Cajal-Retzius cells does not alter hippocampal-dependent behaviors in mice.** Adult (12–14-week-old) mice were subject to (**A**) the open field assay, (**B**) the light–dark box assay, (**C**) the elevated zero maze test, (**D**) the spontaneous Y maze test, and (**E**) the context and cued fear conditioning tests. Data are presented as scatter plots for both female and male mice with all data points shown and error bars representing ± SD. Each data point (N) is an individual animal. Statistical analyses were performed using ordinary one-way ANOVA with Tukey’s *post hoc* test. Download Figure 7-1, TIF file.

## Discussion

Extensive evidence supports the key role of CXCL12 and its receptors in hippocampal development and function (Lu et al., 2002; Li and Ransohoff, 2008; Mithal et al., 2012; Schultheiss et al., 2013; Mimura-Yamamoto et al., 2017; Wu et al., 2017; Abe et al., 2018; Trousse et al., 2019). Multiple studies have also reported *Cxcl12* expression in hippocampal CR cells using a range of techniques (Bagri et al., 2002; Lu et al., 2002; Abe et al., 2018; Hochgerner et al., 2018). However, the functional significance of CR cell-derived CXCL12 in the developing or adult hippocampus remains unresolved. Here, by selectively deleting CXCL12 in hippocampal CR cells, we found that this partial loss does not produce overt changes in hippocampal architecture, adult neurogenesis, or hippocampal-dependent behaviors.

To map *Cxcl12* expression, we used the *Cxcl12-dsRED* reporter mouse line, a validated tool for identifying *Cxcl12*-expressing cells (Ding and Morrison, 2013; Pitt et al., 2015; Das et al., 2019; Chandrasekaran et al., 2022; Lee et al., 2024). Strong reporter signal was detected in CR cells, vasculature, and meninges, consistent with previous findings (Bagri et al., 2002; Lu et al., 2002; Abe et al., 2018; Hochgerner et al., 2018). Notably, only 20–50% of CR cells expressed *Cxcl12*, depending on age, suggesting potential heterogeneity within this population despite a shared cortical hem origin (Takiguchi-Hayashi et al., 2004; Louvi et al., 2007; Gu et al., 2011). A similar pattern of heterogeneous *Cxcl12* expression has also been reported in E18 neocortical CR cells, which are likewise derived from the cortical hem (Iacono et al., 2018), indicating that this variability may emerge early during lineage specification. While prior studies have described *Cxcl12* expression in the early postnatal hippocampus, we show for the first time that it is also expressed in CR cells in the adult hippocampus. However, the proportion and the density of *Cxcl12*-expressing CR cells drop dramatically in adulthood compared with early postnatal ages. The basis and significance of this age-dependent decline remain to be determined.

The preservation of hippocampal structure, neurogenesis, and behavior in the Δ*Np73-Cre; Cxcl12^flox/flox^* knock-out mice raises interesting questions about the necessity of CR cell-derived CXCL12. One key consideration is the efficiency of deletion: only a subset of CR cells loses CXCL12 in this model. The residual CXCL12 may be sufficient to maintain normal hippocampal function. Although we validated *Cxcl12* recombination at P5, Δ*Np73-Cre* is active during embryonic CR cell differentiation (Tissir et al., 2009), and prior studies have shown robust *Cxcl12* expression in embryonic CR cells (Yamazaki et al., 2004). Additional validation at embryonic stages could therefore further strengthen our conclusions. Future studies could also employ alternative Cre lines, such as *Wnt3a-Cre* (Yoshida et al., 2006; Louvi et al., 2007) or *Pde1c-Cre* (Anstotz et al., 2018), which may achieve broader deletion, to assess resulting phenotypes. Another possibility is that other CXCL12-expressing cells—such as endothelial and vascular leptomeningeal cells—compensate for its loss in CR cells. This interpretation is supported by our observation that overall hippocampal CXCL12 protein levels remain unchanged in knock-out mice. These sources are well established (Abe et al., 2018; Hochgerner et al., 2018) and may play a dominant role, especially in adulthood when CR cells are fewer and produce less CXCL12. Redundancy in source may thus buffer against any functional consequences of CR cell-specific loss. Future studies could investigate the effects of CXCL12 deletion from endothelial cells and/or vascular leptomeninges cells on hippocampus development and function. Compensatory signaling may also occur at the receptor level. Previous studies show that CXCR4 expression increases in response to reduced CXCL12 levels (Kolodziej et al., 2008; Schultheiss et al., 2013), and deletion of CXCL12 from dentate gyrus granule neurons leads to pronounced CXCR4 upregulation (Abe et al., 2018). It is plausible that partial CXCL12 loss in CR cells triggers receptor upregulation, thereby maintaining downstream signaling. Notably, CR cells themselves express *Cxcr4* (Stumm et al., 2003; Anstotz et al., 2014; Anstotz and Maccaferri, 2020), raising the possibility of autocrine CXCL12–CXCR4 signaling within CR cells. In this context, loss of CXCL12 may drive compensatory changes within CR cells—such as CXCR4 upregulation—that could further buffer against functional deficits.

Our behavioral analyses corroborate the histological findings: across a battery of hippocampal-dependent tasks, knock-out mice performed similarly to controls. These results suggest that any subtle circuit alterations were insufficient to cause behavioral impairments under baseline conditions. However, stress-inducing or disease-related paradigms may reveal deficits not apparent here. Given the role of CXCL12 in neuroinflammation (Yan et al., 2022; Cambier et al., 2023), future studies should examine the impact of CR cell-specific CXCL12 loss under pathological conditions—such as injury, neurodegeneration, or epilepsy—when neurogenesis and circuit remodeling are more dynamically regulated. This question may be particularly relevant during the first 2 postnatal weeks, when *Cxcl12*-expressing CR cells exhibit preferential survival, suggesting that they may serve distinct developmental or neuroprotective roles during this critical window. Experimental paradigms involving perinatal stress or neuroinflammation could therefore provide valuable insight into the context-dependent functions of *Cxcl12* in CR cells.

In conclusion, our findings demonstrate that partial deletion of CXCL12 from CR cells does not overtly impact hippocampal development, adult neurogenesis, or behavior. These results highlight the complexity and redundancy of chemokine signaling network in the hippocampus and suggest that CR cell-derived CXCL12 may be dispensable under normal physiological conditions. Further studies are needed to assess its role under stress or disease to fully understand its contribution to hippocampal function.

## Synthesis

Reviewing Editor: Nathalie Ginovart, University of Geneva Decisions are customarily a result of the Reviewing Editor and the peer reviewers coming together and discussing their recommendations until a consensus is reached. When revisions are invited, a fact-based synthesis statement explaining their decision and outlining what is needed to prepare a revision will be listed below. The following reviewer(s) agreed to reveal their identity: NONE.

The study addresses an important developmental question, and its results provide valuable insights into the role of the CR-derived Cxcl12 protein in hippocampal development. However, several key points require clarification and additional experimental work before publication can be considered. Specifically, the reviewer suggests expanding the analysis to include critical developmental events such as interneuron programmed cell death and astrocyte proliferation, which are known to interact with Cxcl12 signaling. Furthermore, a more complete description of Cxcr4 protein expression during the postnatal developmental window and the addition of some missing experimental data (e.g., representative images, quantifications, and additional markers of neurogenesis) would strengthen the interpretation of the results.

Please find the reviewer's comments below:

Reviewer:

This work interrogates the role of cajal-retzius cells (CR)-derived Cxcl12 during postnatal hippocampal development. Using a conditional knockout, preventing a subset of CRs to express Cxcl12, different aspects of hippocampal morphogenesis and hippocampal-related behaviours are analysed. It tackles a relevant developmental question, the function of different chemokine sources ensuring proper hippocampal development. Overall, it demonstrates that CR-derived Cxcl12 is postnatally dispensable for hippocampal morphogenesis and therefore for some of its related behaviours.

Despite the quality and the interest of the work, it doesn't address, or highlight, two major developmental events occurring within the first postnatal week: programmed interneuron cell death and astrocytic proliferation. These two cell types express the Cxcl12 receptor Cxcr4 and are known to be susceptible to this chemokine throughout their developmental history. Also, some elements are missing in the main figures and should be added for a more comprehensive interpretation of the results.

Please find below some comments that should be addressed before publication:

A cell-type specific description of Cxcr4 expression during the analysed time window would point towards possible cell-type specific susceptibilities to CR-derived Cxcl12 depletion. Also, a description of Cxcl12 expression before the analysed timepoints and from when it is depleted in ∆Np73+ CRs would increase the background and reasoning behind the focus on postnatal stages. This could be done either by providing literature references or by showing some earlier timepoints of the Cxcl12-DsRed.

As proposed in the discussion, measuring the drop of Cxcl12 between controls and ∆Np73-Cre; Cxcl12flox/flox mutants would reinforce the idea that other sources of Cxcl12 might compensate for CR-derived Cxcl12 expression loss. This could be performed through a western blot on hippocampal protein extractions.

A quantification of total numbers or density of GABAergic neurons and astrocytes by immunolabelling for GAD2 and SOX9 (to complement GFAP immunostaining) respectively during postnatal development would determine the influence of CR-derived Cxcl12 on GABAergic neuron programmed cell death and astrocyte proliferation, two critical events for these populations that take place during the studied time window.

Unlike most CR, Cxcl12 expressing ones, only undergo cell death after the first 10 postnatal days (Figure 1D and E). This reinforces the idea that this population is preserved, over the others, for a developmental reason. Adding a small characterization or a brief discussion on their identity and biological function would reinforce the rationale to study the role of this chemokine during early postnatal development.

∆Np73-Cre; Cxcl12flox/flox representative images in Figure 3C and G, Figure 4C D are required to illustrate the presented quantifications.

The potential role on adult neurogenesis is interesting and a nice highlight of the study. This work characterizes NSCs using an adequate combination of Gfap and Sox2. However, to support the claim on the preservation of adult neurogenesis, adding a Ki67 staining or an EdU pulse analysis would better characterize NSCs proliferation/quiescence and overall proliferation in the dentate gyrus.

## Author Response

We thank the reviewer and editor for their positive feedback and constructive suggestions. In this revised version, we have thoroughly addressed all concerns raised by the reviewer. The new data strengthen the rigor of our analyses and improve the overall quality of the manuscript. Detailed responses to each comment, along with corresponding changes in the text, are provided below (original referee comments are quoted in bold italics, and our responses follow in plain text; bolded text in our responses refers to revised sections in the manuscript). Two versions of the revised manuscript are provided: one with highlighted changes and one clean copy. All page numbers cited in this rebuttal correspond to the highlighted version of the revised manuscript.

Synthesis Statement for Author:

The study addresses an important developmental question, and its results provide valuable insights into the role of the CR-derived Cxcl12 protein in hippocampal development. However, several key points require clarification and additional experimental work before publication can be considered. Specifically, the reviewer suggests expanding the analysis to include critical developmental events such as interneuron programmed cell death and astrocyte proliferation, which are known to interact with Cxcl12 signaling. Furthermore, a more complete description of Cxcr4 protein expression during the postnatal developmental window and the addition of some missing experimental data (e.g., representative images, quantifications, and additional markers of neurogenesis) would strengthen the interpretation of the results.

Reviewer comments:

This work interrogates the role of cajal-retzius cells (CR)-derived Cxcl12 during postnatal hippocampal development. Using a conditional knockout, preventing a subset of CRs to express Cxcl12, different aspects of hippocampal morphogenesis and hippocampal-related behaviours are analysed. It tackles a relevant developmental question, the function of different chemokine sources ensuring proper hippocampal development. Overall, it demonstrates that CR-derived Cxcl12 is postnatally dispensable for hippocampal morphogenesis and therefore for some of its related behaviours.

Despite the quality and the interest of the work, it doesn't address, or highlight, two major developmental events occurring within the first postnatal week: programmed interneuron cell death and astrocytic proliferation. These two cell types express the Cxcl12 receptor Cxcr4 and are known to be susceptible to this chemokine throughout their developmental history. Also, some elements are missing in the main figures and should be added for a more comprehensive interpretation of the results.

Please find below some comments that should be addressed before publication:

A cell-type specific description of Cxcr4 expression during the analysed time window would point towards possible cell-type specific susceptibilities to CR-derived Cxcl12 depletion. Also, a description of Cxcl12 expression before the analysed timepoints and from when it is depleted in ∆Np73+ CRs would increase the background and reasoning behind the focus on postnatal stages. This could be done either by providing literature references or by showing some earlier timepoints of the Cxcl12-DsRed.

We agree that this information is valuable. In the revised manuscript, we have added relevant literature references describing Cxcr4 mRNA and protein expression, as well as Cxcl12 expression, from early embryogenesis through adulthood (Introduction, pages 3-4; Results, page 13).

As proposed in the discussion, measuring the drop of Cxcl12 between controls and ∆Np73-Cre; Cxcl12flox/flox mutants would reinforce the idea that other sources of Cxcl12 might compensate for CR-derived Cxcl12 expression loss. This could be performed through a western blot on hippocampal protein extractions.

As suggested, we performed western blot analysis on hippocampal protein extracts from P10 mice and found that total CXCL12 levels were unchanged in the knockout mice compared to controls (Fig. 2-1; Results, page 14). These findings support the notion that other cellular sources of Cxcl12 may compensate for the loss of CR-derived CXCL12 (Discussion, page 20).

A quantification of total numbers or density of GABAergic neurons and astrocytes by immunolabelling for GAD2 and SOX9 (to complement GFAP immunostaining) respectively during postnatal development would determine the influence of CR-derived Cxcl12 on GABAergic neuron programmed cell death and astrocyte proliferation, two critical events for these populations that take place during the studied time window.

We agree with the reviewer that evaluating potential changes in GABAergic neurons and astrocytes would strengthen our understanding of how Cxcl12 loss from CR cells influences hippocampal development. Accordingly, we performed immunostaining on P20 brain sections for GABA and GAD67 to label GABAergic neurons (Fig. 4) and for GFAP, NFIA, and SOX2 to label astrocytes (Fig. 5). Quantitative analyses revealed no significant differences in the number or distribution of either cell population between control and mutant mice across the CA1 region, hippocampal fissure, dentate gyrus subgranular zone, or hilus. These findings suggest that CR-derived CXCL12 does not play a major role in the migration or survival of GABAergic neurons or in the proliferation of astrocytes (Results, page 16).

Unlike most CR, Cxcl12 expressing ones, only undergo cell death after the first 10 postnatal days (Figure 1D and E). This reinforces the idea that this population is preserved, over the others, for a developmental reason. Adding a small characterization or a brief discussion on their identity and biological function would reinforce the rationale to study the role of this chemokine during early postnatal development.

We thank the reviewer for this insightful comment, which we have incorporated into our interpretation of the results (page 14). We have also expanded the Discussion (page 19) to include a brief section referencing a study that reported similar heterogeneity in Cxcl12 expression among E18 cortical CR cells. While it is tempting to speculate that the selective survival of Cxcl12-expressing CR cells before P10 may reflect distinct functional roles, our detailed characterization suggests this is unlikely under homeostatic conditions. Nevertheless, the potential role of CR-derived CXCL12 in contexts such as perinatal stress or neuroinflammation remains an interesting avenue for future investigation (Discussion, page 21). ∆Np73-Cre; Cxcl12flox/flox representative images in Figure 3C and G, Figure 4C D are required to illustrate the presented quantifications.

As suggested, representative images from all three genotypes have been added (Fig. 3C and G; Fig. 6C-E).

The potential role on adult neurogenesis is interesting and a nice highlight of the study. This work characterizes NSCs using an adequate combination of Gfap and Sox2. However, to support the claim on the preservation of adult neurogenesis, adding a Ki67 staining or an EdU pulse analysis would better characterize NSCs proliferation/quiescence and overall proliferation in the dentate gyrus.

As suggested, we performed Ki67 immunostaining and quantified the number of Ki67⁺ radial glia-like cells. We found no significant differences in the number of proliferating radial glia-like cells between control and mutant mice (Fig. 6D; Results, page 17). These findings further support our conclusion that adult neurogenesis remains unaltered following partial loss of CXCL12 from CR cells.

## References

[B1] Abe P, Wust HM, Arnold SJ, van de Pavert SA, Stumm R (2018) CXCL12-mediated feedback from granule neurons regulates generation and positioning of new neurons in the dentate gyrus. Glia 66:1566–1576. 10.1002/glia.23324 29537098

[B2] Altman J, Bayer SA (1990) Migration and distribution of two populations of hippocampal granule cell precursors during the perinatal and postnatal periods. J Comp Neurol 301:365–381. 10.1002/cne.903010304 2262596

[B7] Anstotz M, Maccaferri G (2020) A toolbox of criteria for distinguishing Cajal-Retzius cells from other neuronal types in the postnatal mouse hippocampus. eNeuro 7:1–21. 10.1523/ENEURO.0516-19.2019 PMC7004485 31907212

[B3] Anstotz M, Cosgrove KE, Hack I, Mugnaini E, Maccaferri G, Lubke JH (2014) Morphology, input-output relations and synaptic connectivity of Cajal-Retzius cells in layer 1 of the developing neocortex of CXCR4-EGFP mice. Brain Struct Funct 219:2119–2139. 10.1007/s00429-013-0627-2 24026287 PMC4223538

[B4] Anstotz M, Huang H, Marchionni I, Haumann I, Maccaferri G, Lubke JH (2016) Developmental profile, morphology, and synaptic connectivity of Cajal-Retzius cells in the postnatal mouse hippocampus. Cereb Cortex 26:855–872. 10.1093/cercor/bhv271 26582498 PMC4712808

[B5] Anstotz M, Lee SK, Neblett TI, Rune GM, Maccaferri G (2018) Experience-Dependent regulation of Cajal-Retzius cell networks in the developing and adult mouse hippocampus. Cereb Cortex 28:672–687. 10.1093/cercor/bhx153 28637318 PMC6059129

[B6] Anstotz M, Lee SK, Maccaferri G (2022) Glutamate released by Cajal-Retzius cells impacts specific hippocampal circuits and behaviors. Cell Rep 39:110822. 10.1016/j.celrep.2022.110822 35584670 PMC9190441

[B8] Bagri A, Gurney T, He X, Zou YR, Littman DR, Tessier-Lavigne M, Pleasure SJ (2002) The chemokine SDF1 regulates migration of dentate granule cells. Development 129:4249–4260. 10.1242/dev.129.18.4249 12183377

[B9] Banisadr G, Podojil JR, Miller SD, Miller RJ (2016) Pattern of CXCR7 gene expression in mouse brain under normal and inflammatory conditions. J Neuroimmune Pharmacol 11:26–35. 10.1007/s11481-015-9616-y 25997895 PMC4831709

[B10] Bifari F, et al. (2017) Neurogenic radial Glia-like cells in meninges migrate and differentiate into functionally integrated neurons in the neonatal cortex. Cell Stem Cell 20:360–373 e367. 10.1016/j.stem.2016.10.020 27889318

[B11] Borrell V, Marin O (2006) Meninges control tangential migration of hem-derived Cajal-Retzius cells via CXCL12/CXCR4 signaling. Nat Neurosci 9:1284–1293. 10.1038/nn1764 16964252

[B12] Cali C, Bezzi P (2010) CXCR4-mediated glutamate exocytosis from astrocytes. J Neuroimmunol 224:13–21. 10.1016/j.jneuroim.2010.05.004 20580441

[B13] Cambier S, Gouwy M, Proost P (2023) The chemokines CXCL8 and CXCL12: molecular and functional properties, role in disease and efforts towards pharmacological intervention. Cell Mol Immunol 20:217–251. 10.1038/s41423-023-00974-6 36725964 PMC9890491

[B14] Causeret F, Moreau MX, Pierani A, Blanquie O (2021) The multiple facets of Cajal-Retzius neurons. Development 148:1–13. 10.1242/dev.199409 34047341

[B15] Chandrasekaran P, et al. (2022) CXCL12 defines lung endothelial heterogeneity and promotes distal vascular growth. Development 149:1–15. 10.1242/dev.200909 PMC9687018 36239312

[B16] Das S, et al. (2019) A unique collateral artery development program promotes neonatal heart regeneration. Cell 176:1128–1142 e1118. 10.1016/j.cell.2018.12.023 30686582 PMC6435282

[B17] Ding L, Morrison SJ (2013) Haematopoietic stem cells and early lymphoid progenitors occupy distinct bone marrow niches. Nature 495:231–235. 10.1038/nature11885 23434755 PMC3600153

[B18] Elorriaga V, Pierani A, Causeret F (2023) Cajal-Retzius cells: recent advances in identity and function. Curr Opin Neurobiol 79:102686. 10.1016/j.conb.2023.102686 36774666

[B19] Elorriaga V, et al. (2025) Differential contribution of P73+ Cajal-Retzius cells and Reelin to cortical morphogenesis. Development 152:1–14. 10.1242/dev.204451 40207459

[B20] Forster E, Zhao S, Frotscher M (2006) Laminating the hippocampus. Nat Rev Neurosci 7:259–267. 10.1038/nrn1882 16543914

[B21] Gilley JA, Yang CP, Kernie SG (2011) Developmental profiling of postnatal dentate gyrus progenitors provides evidence for dynamic cell-autonomous regulation. Hippocampus 21:33–47. 10.1002/hipo.20719 20014381 PMC2895957

[B22] Glaerum IL, et al. (2024) Postnatal persistence of hippocampal Cajal-Retzius cells has a crucial role in the establishment of the hippocampal circuit. Development 151:1–17. 10.1242/dev.202236 PMC10820737 38095282

[B23] Greenbaum A, Hsu YM, Day RB, Schuettpelz LG, Christopher MJ, Borgerding JN, Nagasawa T, Link DC (2013) CXCL12 in early mesenchymal progenitors is required for haematopoietic stem-cell maintenance. Nature 495:227–230. 10.1038/nature11926 23434756 PMC3600148

[B24] Gu X, Liu B, Wu X, Yan Y, Zhang Y, Wei Y, Pleasure SJ, Zhao C (2011) Inducible genetic lineage tracing of cortical hem derived Cajal-Retzius cells reveals novel properties. PLoS One 6:e28653. 10.1371/journal.pone.0028653 22174859 PMC3236758

[B25] Ha S, Tripathi PP, Daza RA, Hevner RF, Beier DR (2020) Reelin mediates hippocampal Cajal-Retzius cell positioning and infrapyramidal blade morphogenesis. J Dev Biol 8:1–18. 10.3390/jdb8030020 PMC7558149 32962021

[B26] Hatami M, Conrad S, Naghsh P, Alvarez-Bolado G, Skutella T (2018) Cell-biological requirements for the generation of dentate gyrus granule neurons. Front Cell Neurosci 12:402. 10.3389/fncel.2018.00402 30483057 PMC6240695

[B27] Hochgerner H, Zeisel A, Lonnerberg P, Linnarsson S (2018) Conserved properties of dentate gyrus neurogenesis across postnatal development revealed by single-cell RNA sequencing. Nat Neurosci 21:290–299. 10.1038/s41593-017-0056-2 29335606

[B28] Hourigan B, Balay SD, Yee G, Sharma S, Tan Q (2021) Capicua regulates the development of adult-born neurons in the hippocampus. Sci Rep 11:11725. 10.1038/s41598-021-91168-5 34083623 PMC8175746

[B29] Iacono G, Mereu E, Guillaumet-Adkins A, Corominas R, Cusco I, Rodriguez-Esteban G, Gut M, Perez-Jurado LA, Gut I, Heyn H (2018) bigSCale: an analytical framework for big-scale single-cell data. Genome Res 28:878–890. 10.1101/gr.230771.117 29724792 PMC5991513

[B30] Karalay O, et al. (2011) Prospero-related homeobox 1 gene (Prox1) is regulated by canonical Wnt signaling and has a stage-specific role in adult hippocampal neurogenesis. Proc Natl Acad Sci U S A 108:5807–5812. 10.1073/pnas.1013456108 21436036 PMC3078392

[B31] Kempermann G, Song H, Gage FH (2015) Neurogenesis in the adult hippocampus. Cold Spring Harb Perspect Biol 7:a018812. 10.1101/cshperspect.a018812 26330519 PMC4563705

[B32] Kolodziej A, Schulz S, Guyon A, Wu DF, Pfeiffer M, Odemis V, Hollt V, Stumm R (2008) Tonic activation of CXC chemokine receptor 4 in immature granule cells supports neurogenesis in the adult dentate gyrus. J Neurosci 28:4488–4500. 10.1523/JNEUROSCI.4721-07.2008 18434527 PMC6670965

[B33] Lee D, Benvie AM, Steiner BM, Kolba NJ, Ford JG, McCabe SM, Jiang Y, Berry DC (2024) Smooth muscle cell-derived Cxcl12 directs macrophage accrual and sympathetic innervation to control thermogenic adipose tissue. Cell Rep 43:114169. 10.1016/j.celrep.2024.114169 38678562 PMC11413973

[B34] Li G, Adesnik H, Li J, Long J, Nicoll RA, Rubenstein JL, Pleasure SJ (2008) Regional distribution of cortical interneurons and development of inhibitory tone are regulated by Cxcl12/Cxcr4 signaling. J Neurosci 28:1085–1098. 10.1523/JNEUROSCI.4602-07.2008 18234887 PMC3072297

[B35] Li M, Ransohoff RM (2008) Multiple roles of chemokine CXCL12 in the central nervous system: a migration from immunology to neurobiology. Prog Neurobiol 84:116–131. 10.1016/j.pneurobio.2007.11.003 18177992 PMC2324067

[B36] Lopez-Bendito G, Sanchez-Alcaniz JA, Pla R, Borrell V, Pico E, Valdeolmillos M, Marin O (2008) Chemokine signaling controls intracortical migration and final distribution of GABAergic interneurons. J Neurosci 28:1613–1624. 10.1523/JNEUROSCI.4651-07.2008 18272682 PMC6671533

[B37] Louvi A, Yoshida M, Grove EA (2007) The derivatives of the Wnt3a lineage in the central nervous system. J Comp Neurol 504:550–569. 10.1002/cne.21461 17701978

[B38] Lu M, Grove EA, Miller RJ (2002) Abnormal development of the hippocampal dentate gyrus in mice lacking the CXCR4 chemokine receptor. Proc Natl Acad Sci U S A 99:7090–7095. 10.1073/pnas.092013799 11983855 PMC124533

[B39] Martinez-Cerdeno V, Noctor SC (2014) Cajal, retzius, and Cajal-Retzius cells. Front Neuroanat 8:48. 10.3389/fnana.2014.00048 24987337 PMC4060955

[B40] Mercier F, Arikawa-Hirasawa E (2012) Heparan sulfate niche for cell proliferation in the adult brain. Neurosci Lett 510:67–72. 10.1016/j.neulet.2011.12.046 22230891

[B41] Meyer G, Gonzalez-Arnay E, Moll U, Nemajerova A, Tissir F, Gonzalez-Gomez M (2019) Cajal-Retzius neurons are required for the development of the human hippocampal fissure. J Anat 235:569–589. 10.1111/joa.12947 30861578 PMC6704247

[B42] Mimura-Yamamoto Y, Shinohara H, Kashiwagi T, Sato T, Shioda S, Seki T (2017) Dynamics and function of CXCR4 in formation of the granule cell layer during hippocampal development. Sci Rep 7:5647. 10.1038/s41598-017-05738-7 28717168 PMC5514042

[B43] Mithal DS, Banisadr G, Miller RJ (2012) CXCL12 signaling in the development of the nervous system. J Neuroimmune Pharmacol 7:820–834. 10.1007/s11481-011-9336-x 22270883 PMC4526243

[B44] Ogawa M, Miyata T, Nakajima K, Yagyu K, Seike M, Ikenaka K, Yamamoto H, Mikoshiba K (1995) The reeler gene-associated antigen on Cajal-Retzius neurons is a crucial molecule for laminar organization of cortical neurons. Neuron 14:899–912. 10.1016/0896-6273(95)90329-1 7748558

[B45] Pahle J, et al. (2020) Selective inactivation of reelin in inhibitory interneurons leads to subtle changes in the dentate gyrus but leaves cortical layering and behavior unaffected. Cereb Cortex 30:1688–1707. 10.1093/cercor/bhz196 31667489 PMC7132935

[B46] Pitt LA, et al. (2015) CXCL12-Producing vascular endothelial niches control acute T cell leukemia maintenance. Cancer Cell 27:755–768. 10.1016/j.ccell.2015.05.002 26058075 PMC4461838

[B47] Ramezanidoraki N, et al. (2023) Activation of the PI3K/AKT/mTOR pathway in Cajal-Retzius cells leads to their survival and increases susceptibility to kainate-induced seizures. Int J Mol Sci 24:1–21. 10.3390/ijms24065376 PMC10048971 36982451

[B48] Riva M, et al. (2023) Aberrant survival of hippocampal Cajal-Retzius cells leads to memory deficits, gamma rhythmopathies and susceptibility to seizures in adult mice. Nat Commun 14:1531. 10.1038/s41467-023-37249-7 36934089 PMC10024761

[B49] Schultheiss C, et al. (2013) CXCR4 prevents dispersion of granule neuron precursors in the adult dentate gyrus. Hippocampus 23:1345–1358. 10.1002/hipo.22180 23929505

[B50] Simon R, et al. (2012) A dual function of Bcl11b/Ctip2 in hippocampal neurogenesis. EMBO J 31:2922–2936. 10.1038/emboj.2012.142 22588081 PMC3395096

[B51] Stumm RK, Zhou C, Ara T, Lazarini F, Dubois-Dalcq M, Nagasawa T, Hollt V, Schulz S (2003) CXCR4 regulates interneuron migration in the developing neocortex. J Neurosci 23:5123–5130. 10.1523/JNEUROSCI.23-12-05123.2003 12832536 PMC6741192

[B52] Takiguchi-Hayashi K, Sekiguchi M, Ashigaki S, Takamatsu M, Hasegawa H, Suzuki-Migishima R, Yokoyama M, Nakanishi S, Tanabe Y (2004) Generation of reelin-positive marginal zone cells from the caudomedial wall of telencephalic vesicles. J Neurosci 24:2286–2295. 10.1523/Jneurosci.4671-03.2004 14999079 PMC6730420

[B53] Tissir F, Wang CE, Goffinet AM (2004) Expression of the chemokine receptor Cxcr4 mRNA during mouse brain development. Brain Res Dev Brain Res 149:63–71. 10.1016/j.devbrainres.2004.01.002 15013630

[B54] Tissir F, Ravni A, Achouri Y, Riethmacher D, Meyer G, Goffinet AM (2009) Deltanp73 regulates neuronal survival in vivo. Proc Natl Acad Sci U S A 106:16871–16876. 10.1073/pnas.0903191106 19805388 PMC2757832

[B55] Trousse F, Jemli A, Silhol M, Garrido E, Crouzier L, Naert G, Maurice T, Rossel M (2019) Knockdown of the CXCL12/CXCR7 chemokine pathway results in learning deficits and neural progenitor maturation impairment in mice. Brain Behav Immun 80:697–710. 10.1016/j.bbi.2019.05.019 31100368

[B56] van Bruggen R, Patel ZH, Wang M, Suk TR, Rousseaux MWC, Tan Q (2023) A versatile strategy for genetic manipulation of Cajal-Retzius cells in the adult mouse hippocampus. eNeuro 10:1–23. 10.1523/ENEURO.0054-23.2023 PMC10585607 37775311

[B57] Vilchez-Acosta A, Manso Y, Cardenas A, Elias-Tersa A, Martinez-Losa M, Pascual M, Alvarez-Dolado M, Nairn AC, Borrell V, Soriano E (2022) Specific contribution of Reelin expressed by Cajal-Retzius cells or GABAergic interneurons to cortical lamination. Proc Natl Acad Sci U S A 119:e2120079119. 10.1073/pnas.2120079119 36067316 PMC9477240

[B58] Wu PR, Cho KKA, Vogt D, Sohal VS, Rubenstein JLR (2017) The cytokine CXCL12 promotes basket interneuron inhibitory synapses in the medial prefrontal cortex. Cereb Cortex 27:4303–4313. 10.1093/cercor/bhw230 27497284 PMC6410508

[B59] Yamazaki H, Sekiguchi M, Takamatsu M, Tanabe Y, Nakanishi S (2004) Distinct ontogenic and regional expressions of newly identified Cajal-Retzius cell-specific genes during neocorticogenesis. Proc Natl Acad Sci U S A 101:14509–14514. 10.1073/pnas.0406295101 15452350 PMC521974

[B60] Yan Y, Su J, Zhang Z (2022) The CXCL12/CXCR4/ACKR3 response axis in chronic neurodegenerative disorders of the central nervous system: therapeutic target and biomarker. Cell Mol Neurobiol 42:2147–2156. 10.1007/s10571-021-01115-1 34117967 PMC11421623

[B61] Yoshida M, Assimacopoulos S, Jones KR, Grove EA (2006) Massive loss of Cajal-Retzius cells does not disrupt neocortical layer order. Development 133:537–545. 10.1242/dev.02209 16410414

[B62] Yu DX, Marchetto MC, Gage FH (2014) How to make a hippocampal dentate gyrus granule neuron. Development 141:2366–2375. 10.1242/dev.096776 24917496

[B63] Zhu Y, Murakami F (2012) Chemokine CXCL12 and its receptors in the developing central nervous system: emerging themes and future perspectives. Dev Neurobiol 72:1349–1362. 10.1002/dneu.22041 22689506

