# Supplementary figures and images for: Partial Deletion of Cxcl12 from Hippocampal Cajal–Retzius Cells Does Not Disrupt Dentate Gyrus Development or Neurobehaviors
Source: eNeuro. 2026 Jan 7;13(1):ENEURO.0245-25.2025. doi: 10.1523/ENEURO.0245-25.2025 (PMC12803706; doi:10.1523/ENEURO.0245-25.2025)

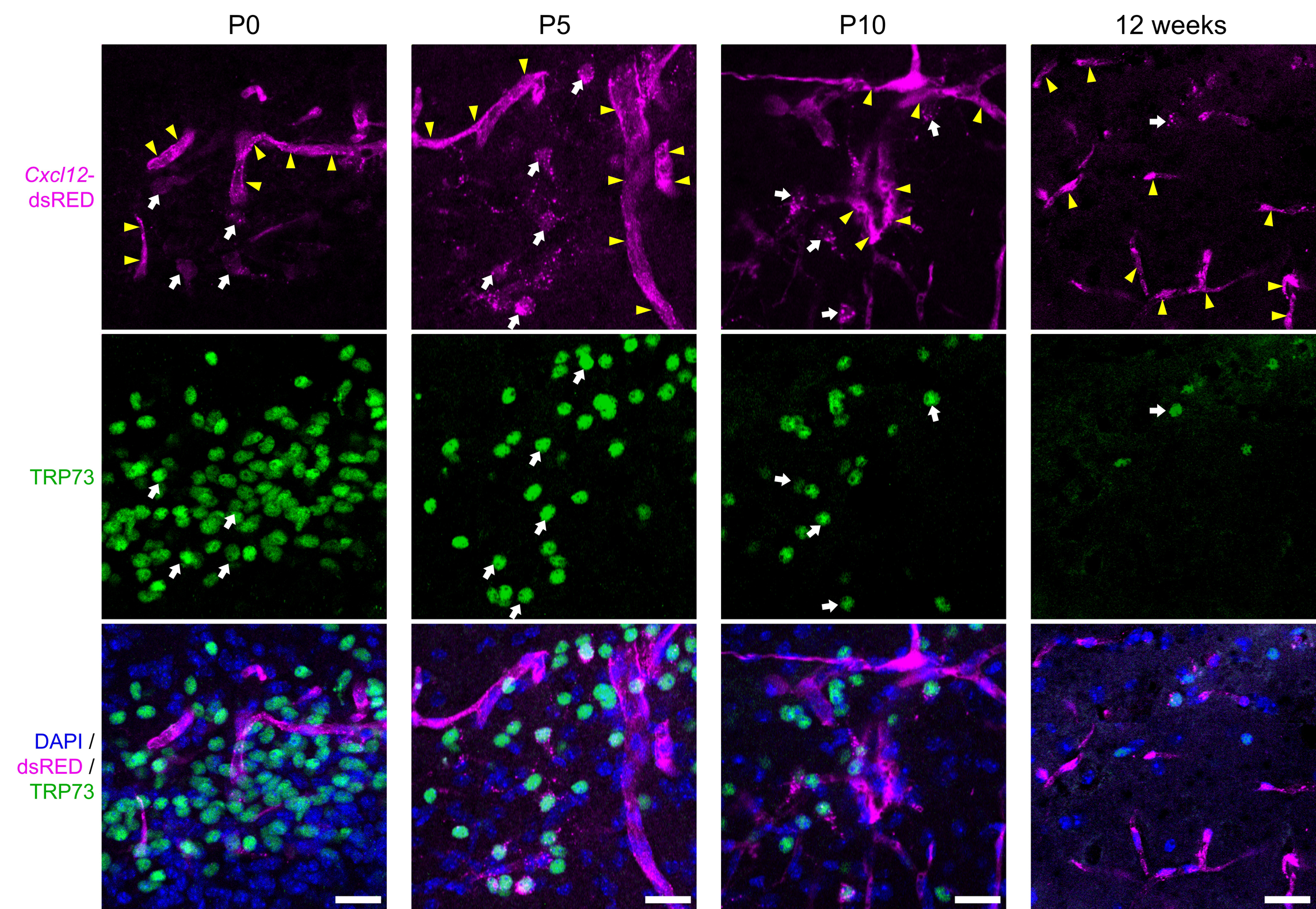

Supplement: Figure 1-2 — Cxcl12 expression in Cajal-Retzius cells and the vasculature in the postnatal hippocampus. Representative confocal images showing co-immunostaining for dsRED and Cajal-Retzius (CR) cell marker TRP73 in the hippocampus at postnatal day (P) 0, P5, P10, and 12 weeks. Yellow arrowheads indicate the vasculature. White arrows indicate TRP73+ CR cells. Scale bars, 25 µm. Download Figure 1-2, TIF file. [file eneuro-13-ENEURO.0245-25.2025-s002.tif]

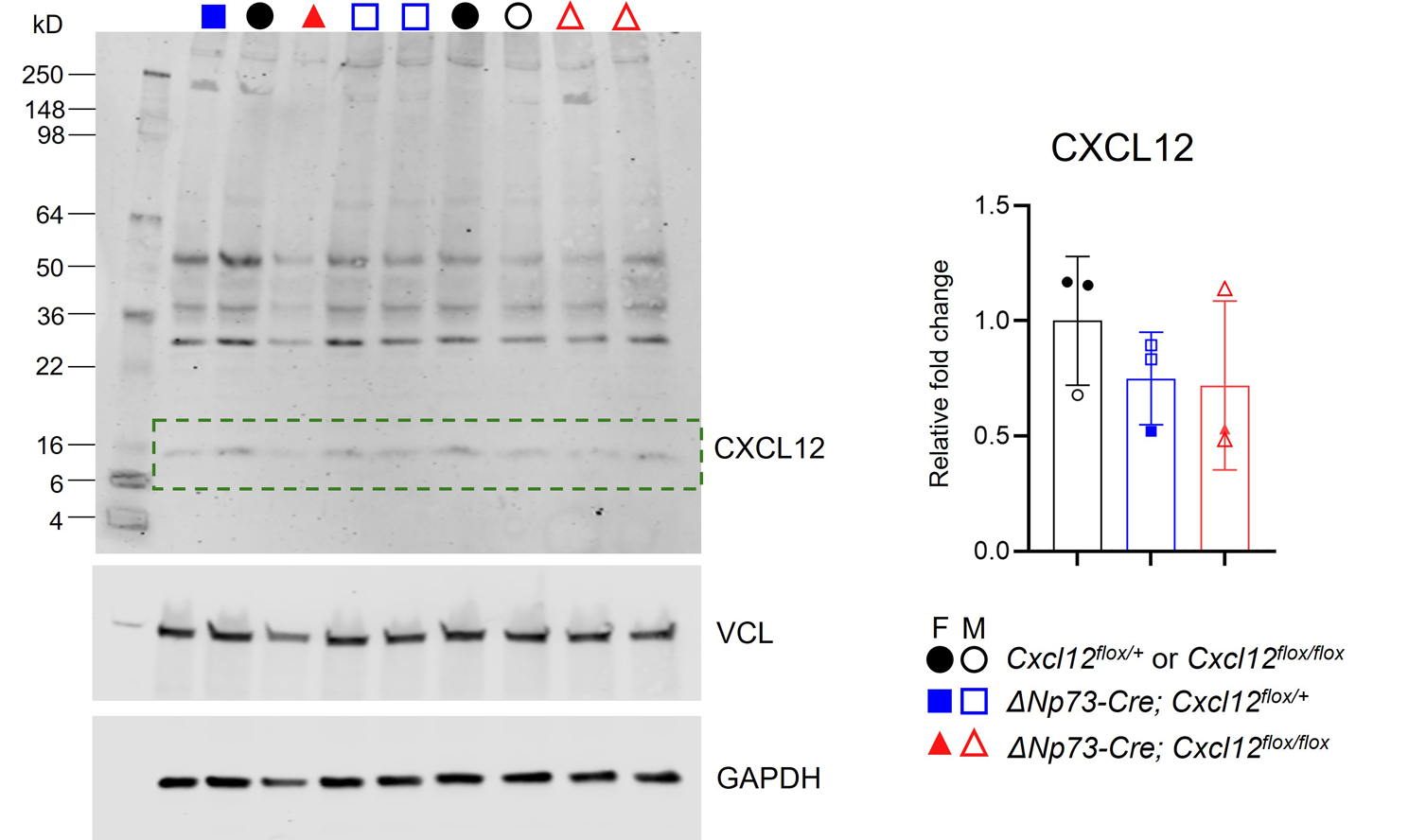

Supplement: Figure 2-1 — Partial deletion of CXCL12 in Cajal-Retzius cells does not alter hippocampal CXCL12 protein levels. Immunoblot showing CXCL12 protein in whole hippocampal lysates from postnatal day (P) 10 control and ΔNp73-Cre; Cxcl12flox/flox mice. Vinculin (VCL) and GAPDH serve as loading controls. Quantification of CXCL12 levels normalized to loading controls is shown at right. Download Figure 2-1, TIF file. [file eneuro-13-ENEURO.0245-25.2025-s003.tif]

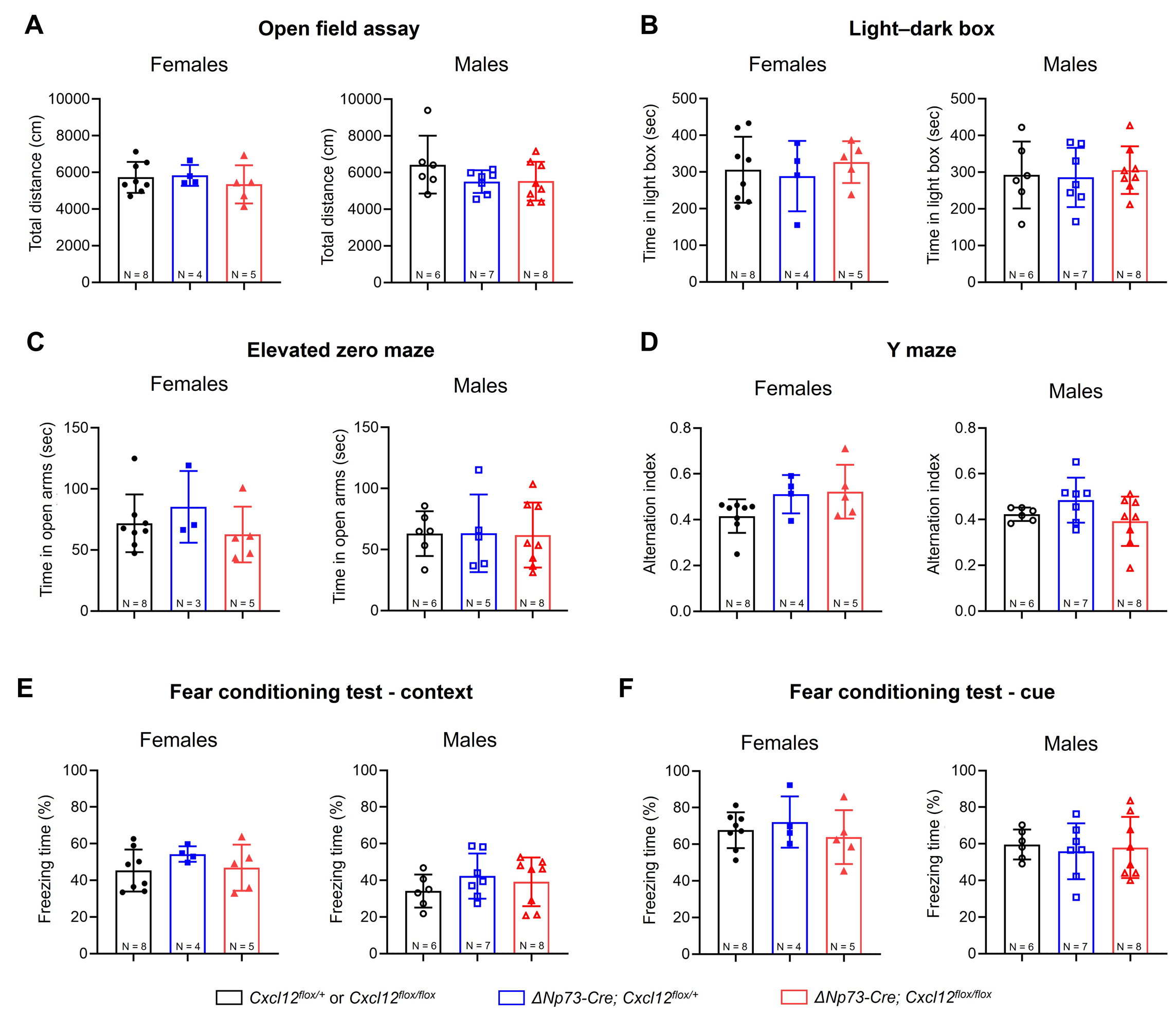

Supplement: Figure 7-1 — Partial deletion of CXCL12 in Cajal-Retzius cells does not alter hippocampal-dependent behaviors in mice. Adult (12–14-week-old) mice were subject to (A) the open field assay, (B) the light–dark box assay, (C) the elevated zero maze test, (D) the spontaneous Y maze test, and (E) the context and cued fear conditioning tests. Data are presented as scatter plots for both female and male mice with all data points shown and error bars representing ± SD. Each data point (N) is an individual animal. Statistical analyses were performed using ordinary one-way ANOVA with Tukey’s post hoc test. Download Figure 7-1, TIF file. [file eneuro-13-ENEURO.0245-25.2025-s004.tif]
